# Supplementary material for: Dietary restriction modulates ultradian rhythms and autocorrelation properties in mice behavior
Source: Commun Biol. 2024 Mar 9;7:303. doi: 10.1038/s42003-024-05991-3 (PMC10925031; doi:10.1038/s42003-024-05991-3)
Supplement: Supplementary file 2 — Supplementary Information [file 42003_2024_5991_MOESM2_ESM.pdf]

Supplementary Information

Index:

Supplementary Note 1. Overview of the GaMoSEC: 5-step wavelet approach for rhythm detection and characterization 2

Supplementary Note 2: Criteria for detection of ultradian rhythms 5

Supplementary Note 3: Characterization of behavioral rhythms and their modulation by the imposed feeding paradigms. 10

Supplementary Note 4: Scaling in behavioral time series estimation 26

Supplementary Note 5: Genetic differences in timing of food consumption seems not related to inter-individual differences in the expression of 12h-rhythms. 34

Supplementary Note 7: Modulation of short- and long-range autocorrelation properties induced by the feeding paradigm over time. 41

Supplementary Note 8: Correlation matrix and scatter plots between variables 43

Supplementary References 46

Supplementary Note 1. Overview of the GaMoSEC: 5-step wavelet approach for rhythm detection and characterization

We developed a 5-step wavelet approach to assess food-intake and wheel running behavioral patterns of mice that were first fed *ad-libitum* and then switched to specific feeding paradigms (see main text). In this 5-step wavelet approach each behavioral time series is sequentially analyzed first with four different wavelet analyses, allowing detection and characterization of embedded rhythmic behavior, followed by a fifth wavelet method that allows estimation of coherence and phase relationships between different behavioral time series. Note that since wavelets are temporally localized, with this approach we can detect when specific changes in dynamics of behavior occurred and characterize these changes using acrophases and power (i.e. strength) of the rhythm. Detailed description of each method and conceptual framework used in the 5-step wavelet approach is provided in [1, 2].

First, the time series were analyzed using a Gaussian continuous wavelet transform (cwt). This transform highlights localized singularities and fluctuations in time (Supplementary Figure 1b, x-axis) at different time scales (Supplementary Figure 1b, y-axis). Herein, a singularity represents the transition between behavioral states (not-feeding to feeding, or not-running to wheel running) at a given time scale. Moreover, the regular intervals between these maxima (red) and minimum (blue) vertical lines at a given time scale is indicative of periodicity. Interestingly, these vertical lines seemingly repetitively bifurcate in a fractal-like manner for decreasing time scales (see example in Figure 1 and Supplementary Figure 1 at the 12h scale). These bifurcations indicate not only that more than one rhythm is present, but also the complex, highly variable nature of these time series. One of the virtues of the Gaussian cwt is that it provides sharp localization in time, however, given the uncertainty

principle this is in detriment of localization in frequency in the case of periodic data, as observable in the notorious leaking (vertical lines) shown in the corresponding Gaussian cwt plot (Supplementary Figure 1 b) over a broad range of scales.

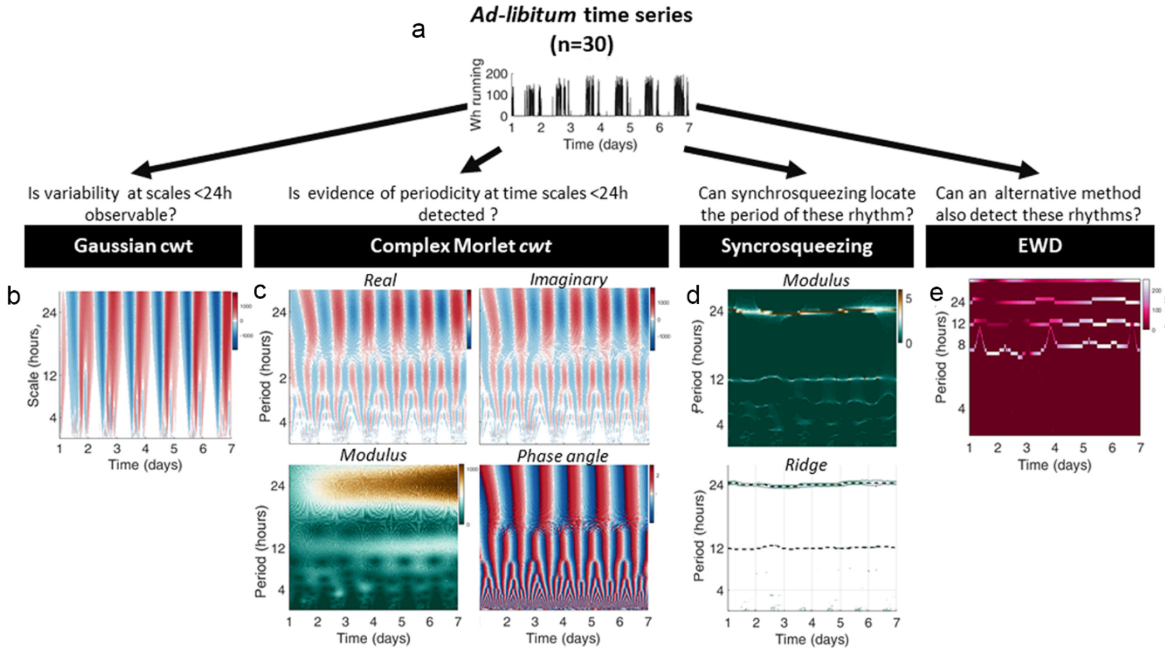

Supplementary Figure 1. Wavelet approach for detection and characterization of animal behavioral patterns and rhythm. a) Example analysis of a wheel running time series for a mice feed ad-libitum during the first 7-days of testing analyzed with the 5-step wavelet approach (only the first 4-steps are shown). **b**) As a first step this time series was analyzed with a first order Gaussian wavelet transform. Plot showed evidence of periodicity (regular spaced alternating red and blue vertical lines) and high variability (i.e. bifurcation-like pattern) in the example time series. **c**) As a second step, a complex Morlet cwt was performed on the example times series to assess periodic behavior. Real, imaginary, modulus and phase angle of the wavelet coefficients are shown. Evidence of rhythmicity at periods around the 24 and 12h time scale is observed. **d**) As a third step, to improve period estimation of rhythms. Ridge (back line) indicates the period associated with maximum coefficients. Note that the rhythms detected present periods of 24h and 12h. **e**) The fourth step consists in verifying results with an independent wavelet analysis, the Empirical Wavelet Decomposition (EWD). Note that if only one horizontal band is observable at the 24h scale with Synchrosqueezing and EWD then the series is classified as absence of evidence for ultradian rhythms. Contrarily, if a second horizontal band is observable in both analyses at the 12h scale then it is considered to present evidence of a 12h ultradian rhythm. See detailed explanation of plot in text.[1]

Once high variability and periodicity was evidenced with the Gaussian cwt, as a second step, behavioral time series were analyzed using a complex Morlet cwt (Supplementary Figures 1 c). Given that the resulting coefficients of this wavelet are complex, four different plots help characterize the time series, namely the real and the imaginary parts, the modulus and the phase angle of the coefficients. Note that the plot of the real part of the complex Morlet cwt coefficients (Supplementary Figures 1 c, top left panel) presents vertical lines with a fractal-like bifurcations pattern similar to the Gaussian cwt (Supplementary Figure 1 b). This bifurcation-like pattern is also evident in the imaginary part and phase angle plots of the cwt coefficients. Moreover, regular intervals between maximum (red) and minimum (blue) vertical lines are also observed at specific time scales (in the example, most notoriously at 24h and 12h time scales) for the real coefficients. Consistent with aperiodicity, the modulus plot presents a white/brown horizontal for time scales around 24 and 12h (Supplementary Figure 1 c, bottom left panel).

To improve period localization and to estimate the relative strength of the observed rhythm, the third step, Wavelet Synchrosqueezing analysis, was performed on behavioral time series (Supplementary Figure 1 e). This analysis provides highly localized frequency estimates. Moreover, maximum values of coefficients are connected by a ridge (black dotted line, Supplementary Figure 1 e), indicating the frequency (1/period, x-axis) of strongest periodic behavior found localized in time (y-axis). In the figure only the first two ridges (black dotted line, Supplementary Figure 1e) were plotted in order to improve visualization. Noteworthy when rhythmicity is present the squared modulus of the Synchrosqueezing coefficient at a given scale (e.g. 24, 12h) is indicative of the power, or strength of these rhythms at the time point. The period of the rhythms is confirmed by the fourth step, an independent analysis, Empirical Wavelet Decomposition (Supplementary Figure 1 d).

Lastly, for characterizing phase relationships and coherence between both behavioral time series obtained from each animal, the last step, Wavelet Coherence was performed (see details Supplementary Note 3).

Supplementary Note 2: Criteria for detection of ultradian rhythms

The GaMoSEC, 5-step wavelet approach was specifically used to determine whether evidence of 24h- and 12h- rhythms was present in food-intake and wheel running time series during each of 5 days periods through the experimental, as shown in Figure 2 a-j (main text). A schematic representation of the decision tree used in this process is shown in Supplementary Figure 2.

As described in the previous section, the first step is to evidence variability at time scales below 24h using a Gaussian wavelet. If variability is not present (Supplementary Figure 2 a), the time series is immediately classified as not presenting evidence of ultradian rhythms. If variability is present, the modulus of the Morlet cwt provides the first approximation of whether there is evidence of periodicity at a specific time scale as a band of positive coefficients (example not shown in Supplementary Figure 2, see Supplementary Figure 1c), while EWD provides additional evidence (Supplementary Figures 2d,e and 3a,b). Note that for EWD positive coefficients are localized at a particular scale (y-axis) representing the period of the oscillation as horizontal bands that can be more or less defined or localized (Supplementary Figures 2d,e). Thus, at the scale representing the period of the UR, if positive coefficients are observed in >75% of the data points it was considered that EWD provided evidence towards the presence of the rhythm (Supplementary Figure 2e, see also Fig. 3a,b). To provide additional evidence that this rhythm is localized in frequency wavelet Synchrosqueezing is performed. If a rhythm is observed with localized frequency (i.e. more than ridge was present in >75% of data points, see example), then it was considered that there was sufficient evidence supporting the presence of the rhythm of a specific period (Supplementary Figure 2f). If the 5-day segment of the time series under analysis fails to comply with any of the above criterias, it is classified as not presenting sufficient evidence of rhythm (Supplementary Figures 2 a,d).

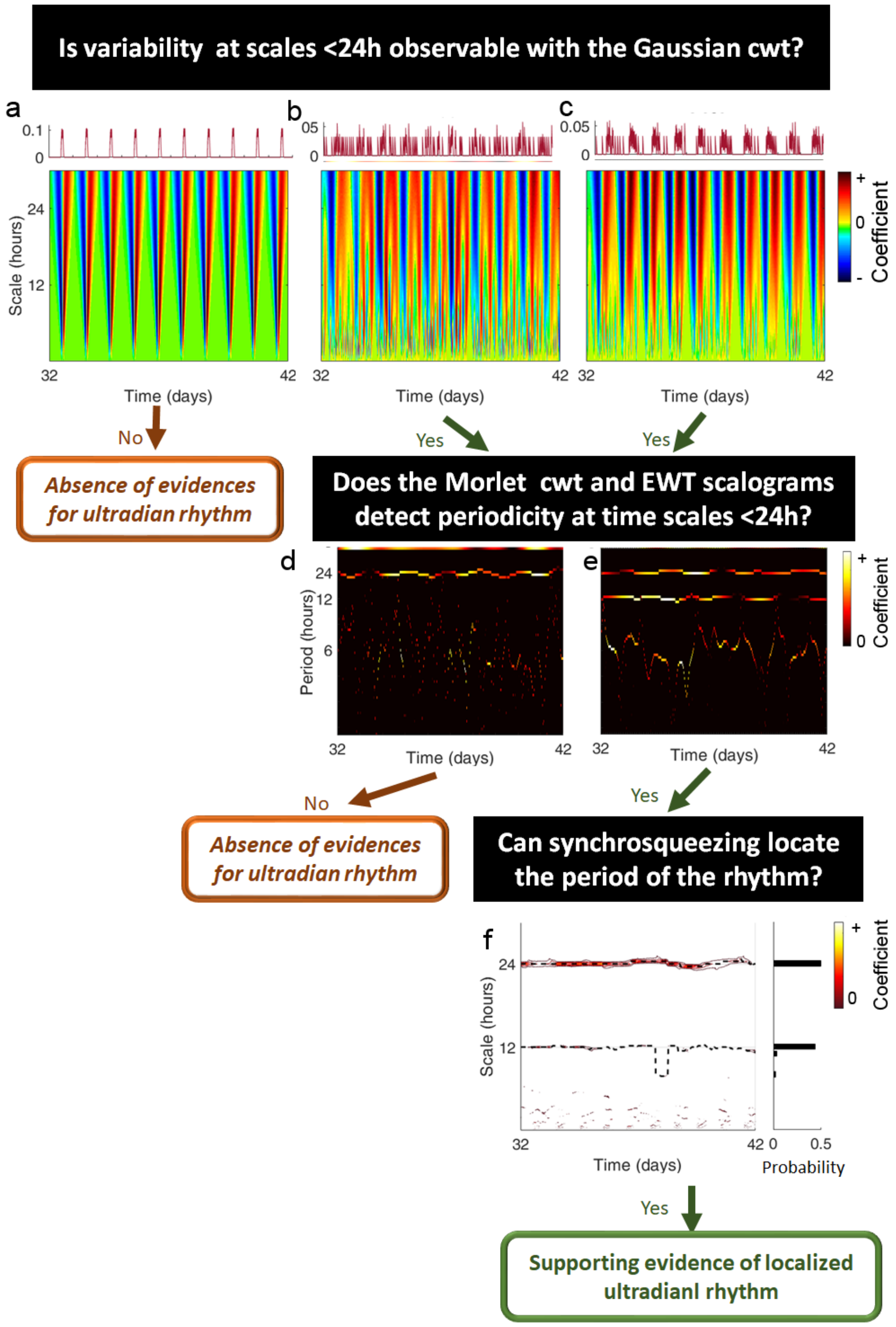

Supplementary Figure 2. Classification criteria for establishing evidence of ultradian rhythms in behavioral time series. Decision tree for classifying time series as either not showing evidence of ultradian rhythm (green) or presenting evidence of ultradian rhythm (orange). **a-c**) Example analysis of food-intake time series (top panels in dark red) for the last ten days of testing from 3 animals are shown. As a first step, a first order Gaussian wavelet transform was performed, if no evidence of high variability was observed for time scaled below 24h then the time series was classified as absent of evidence for any ultradian rhythms. If evidence for high variability was observed, the time series was then analyzed using Morlet cwt (scalogram not shown), followed by Empirical Wavelet Decomposition (EWD, shown in figure panels **d,e**), if only the 24h frequency band is observable the series was classified as absent of evidence for ultradian rhythms. If a second frequency band is observable, Synchrosqueezing (**f**) was performed in order to localize in frequency the ultradian rhythm.

To highlight the capability of Synchrosqueezing to detect localized frequencies Supplementary Figure 3c shows the probability distribution of the ridges detected in the behavioral time series, by the end of the experiment. Note that the first two ridges of the wavelet Synchrosqueezing predominately detect 24h and 12h rhythm (Supplementary Figure 3c, see Table 1, main text). Recall that when rhythmicity is present the squared modulus of the Synchrosqueezing coefficients at a given scale (e.g. 24, 12h) is indicative of the power, or strength of these rhythms at the time point.

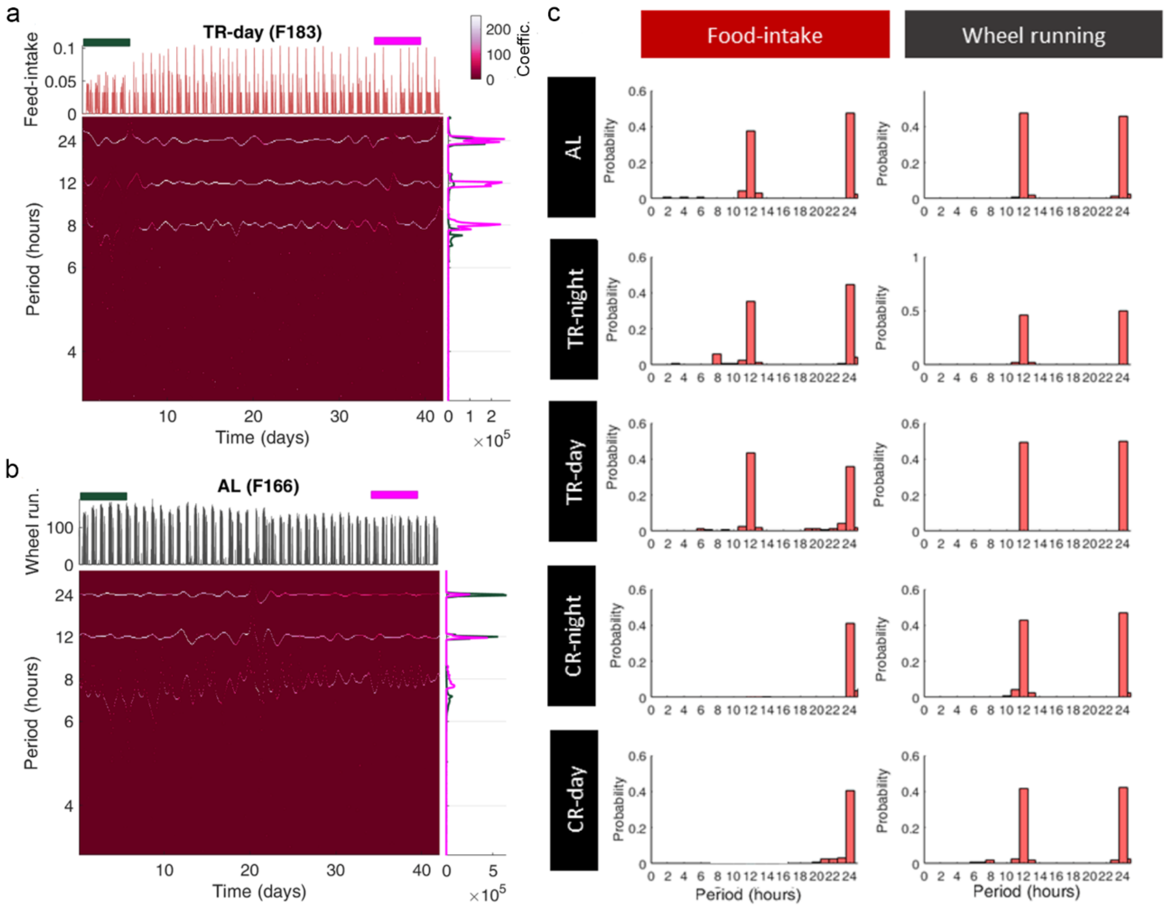

Supplementary Figure 3. Examples of Empirical Wavelet Decomposition (EWD) and probability of distribution of ridges detection using wavelet synchrosqueezing. Two examples of EWD applied to **a**) food-intake and **b**) wheel-running time series. Sum of coefficients for 5-days periods from the first and last-week of experimentation are shown on the right-hand inset in green and magenta lines, respectively. Localized peaks indicate the presence of a rhythm of a specific period. **c**) The probability of detecting a ridge associated with a behavioral rhythm of a given period is shown. For visualization purposes, estimates were performed using only the first two ridges, except for the food-intake CR-night and CR-day time series where only the first ridge is displayed. Synchrosqueezing data from the last 10 days of testing is shown in each panel. Note that predominantly only two period values are observed, namely the 24h and 12h- rhythms.

Comparison between complementary methodology for analysis of dynamical behavioral changes induced by feeding paradigm are shown in Supplementary Figures 4 and 5.

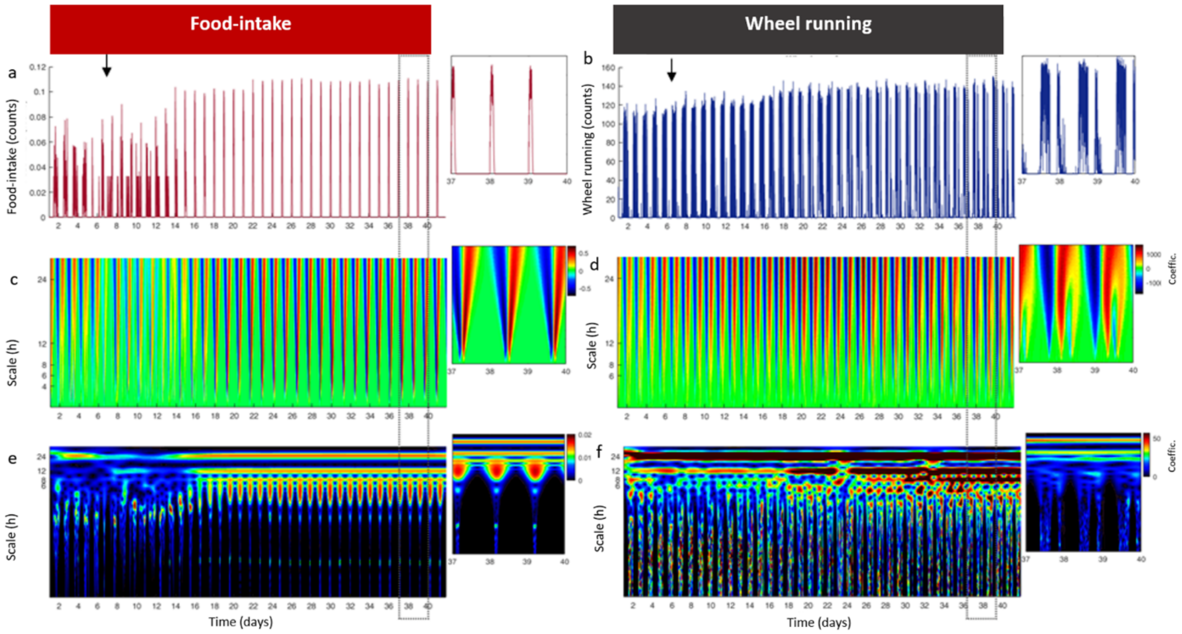

Supplementary Figure 4: Detailed example of time series analysis of a mouse that was switched from an ad-libitum to a CR-day feeding paradigm during testing. Loss of complexity in food-intake time series (**a**) associated with the shift in feeding paradigm from ad libitum feeding to daytime caloric restriction (CR-day) is clearly observable in with both the **c**) Gaussian cwt and complex **e**) Morse cwt (modulus is shown). Compare with the complex pattern observed throughout the experiment in wheel running behavior (**b**), with both wavelet transforms (**d,f**). Note also the clear bifurcation pattern denoting the 12 h ultradian rhythms (UR).

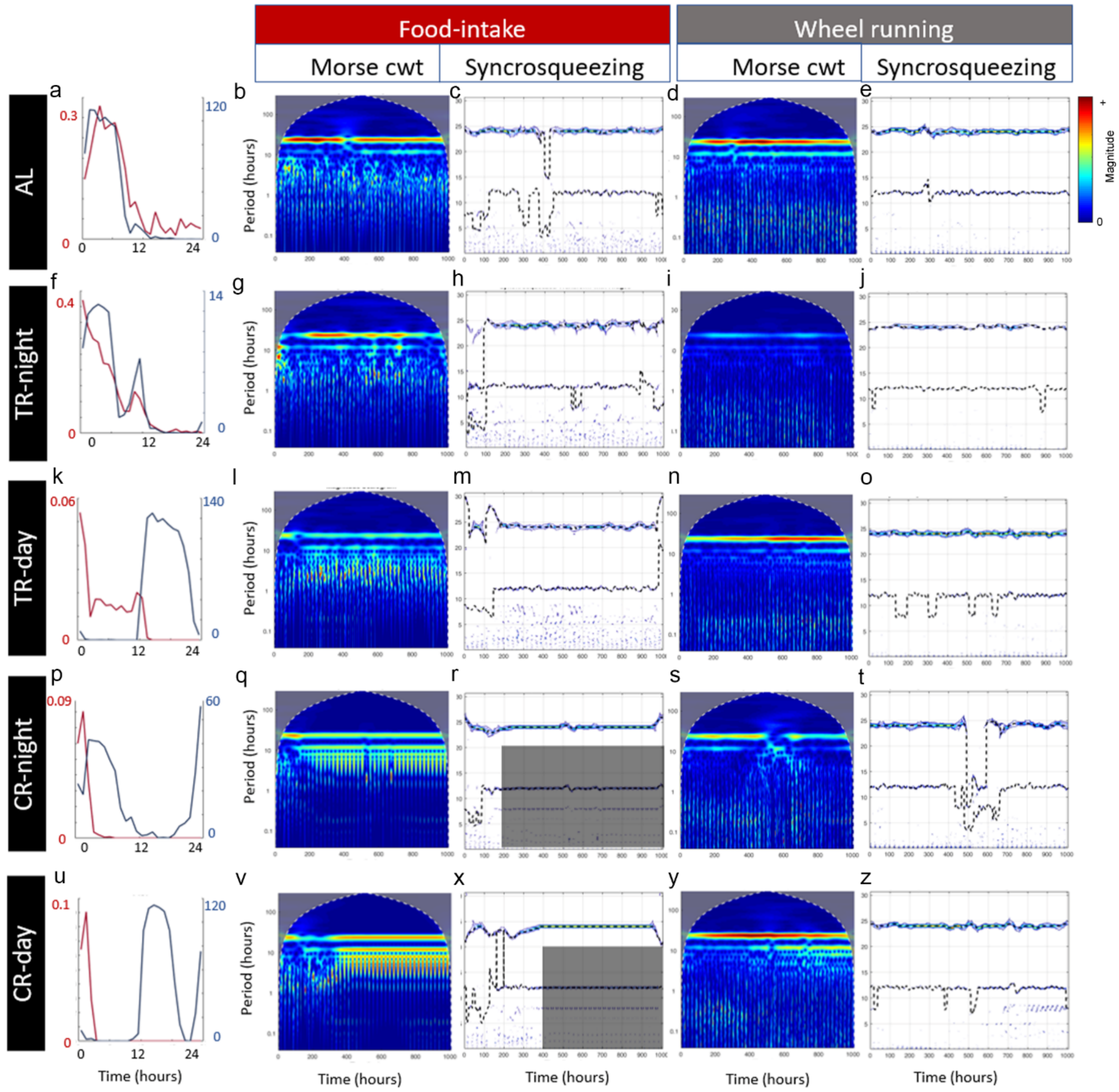

Supplementary Figure 5. Comparison between complementary methodology in time series analysis. a, f, k, p, u) Mean values of food-intake and wheel running over the last 10-days of testing (similar to Supplementary Figure 4 in [3]). In the example time series 2 peaks in wheel running are particularly observable wheel running time series in CR. The modulus of the complex Morse cwt [2] estimated on example **b, g, l, q, v**) food-intake and **d, i, n, s, y**) wheel running time series. **c, e, h, j, m, o, r, t, x, z**) the corresponding wavelet Synchrosqueezing analysis performed on the same time series. Note in both analysis regions of weakness of rhythms are observable, in particular in the wheel running time series of CR-night. Black box in the plots of the wavelet Syncrosqueezing analysis of food-intake time series of CR mice corresponds to scales that lack variability at scales below the 24h scale thus is not considered in analysis according to decision tree shown in Supplementary Figure 4.

Supplementary Note 3: Characterization of behavioral rhythms and their modulation by the imposed feeding paradigms.

Once presence of a rhythm in a behavioral time series is detected with GaMoSEC two main rhythm characterizations were performed throughout the 42-day time, namely acrophase and power. As described in the Methods section of the main text, acrophase was estimated from the real part of the complex Morlet cwt. Supplementary Figures 6 d and e shows scalograms of the real coefficient estimated for food-intake and wheel running time series of a mouse that remained fed *ad libitum* throughout the experiment (Supplementary Figure 6 a-c). The 24h and 12h time scales are indicated with dotted lines. These coefficients are also shown plotted as a function of time in panels g-i and j-l, respectively. Note that, since the time series is periodic at the corresponding time scales, the coefficients are also periodic. In the case of the 24h-rhythm, the time of each peak value represents the acrophase. Since the acrophase potentially can change over time associated with experimental treatment, acrophase was estimated for each day of experimentation (Supplementary Figure 6 l-n). Similarly, the time of day of first and second daily peaks in the 12h rhythm is also estimated using the temporal localization of peaks (Supplementary Figure 6 l-n).

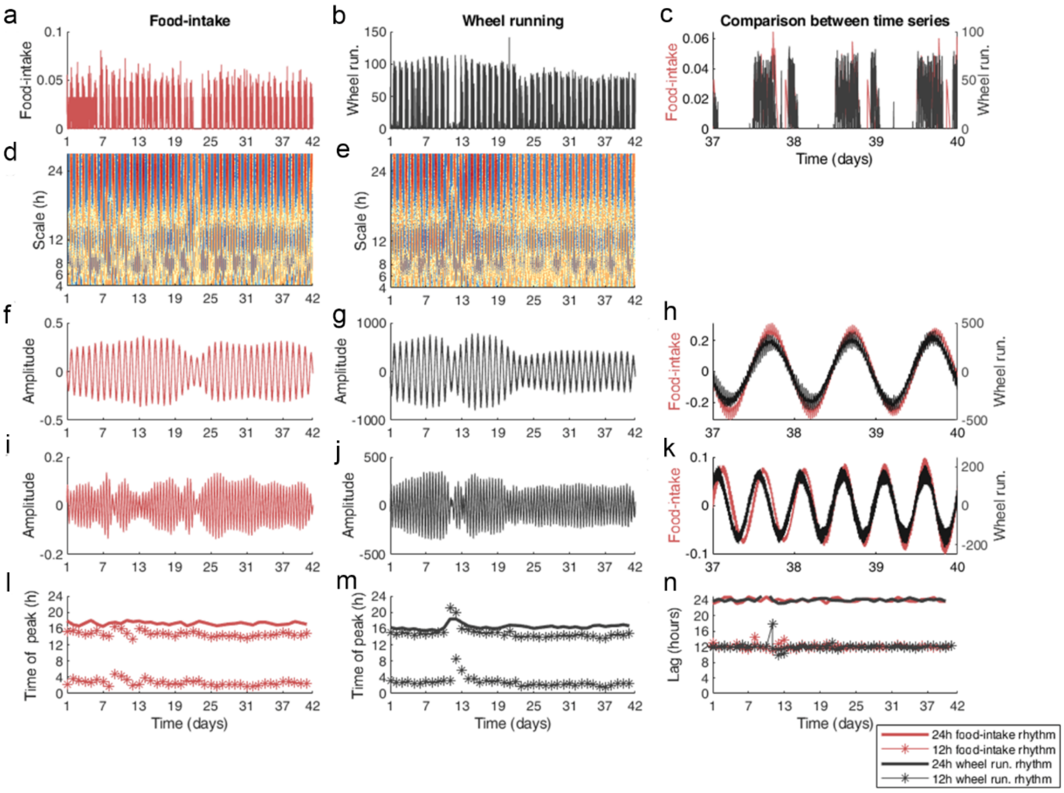

Supplementary Figure 6. Graphical representation of estimation of acrophase and peak estimation in food-intake and wheel running time series. a-c) Examples of food-intake (red) and wheel running (blue) time series from a mouse fed *ad libitum* throughout the experiment. **d,e**) Scalograms of the real part of the wavelet coefficients estimated with a complete Morlet wavelet for time series “a” and “b”. The x-axis represents time (days of experimentation) and the y-axis indicates the scale of the wavelet used (from 6 min to 30 h). Red indicates positive values while blue indicates negative amplitude values, and green approximately 0. Dotted black lines indicate the 24 and 12h scale used in analysis shown in the following panels. Wavelet coefficients obtained at the **f-h**) 24h and **i-k**) 12h scales as a function of time. **l-n**) Peaks in the wavelet coefficients estimated daily are shown for each time scale. ** Level of synchronization between animals can be estimated for each time scale (i.e. 24h or 12h) be calculating the correlation coefficient between the real part of the Morlet estimated for each animal [1].

The fifth step of the GaMoSEC 5-step wavelet approach is aimed at understanding relations between the different time series measured. Herein, wavelet coherence was estimated between the food-intake (Supplementary Figure 7 a) and wheel running (Supplementary Figure 7 b) time series of the individual. Phase relationships between variables at a given temporal scale are also estimated (Supplementary Figure 7 c, arrows), and acrophase estimations are shown (solid lines , Supplementary Figures 7 a,b).

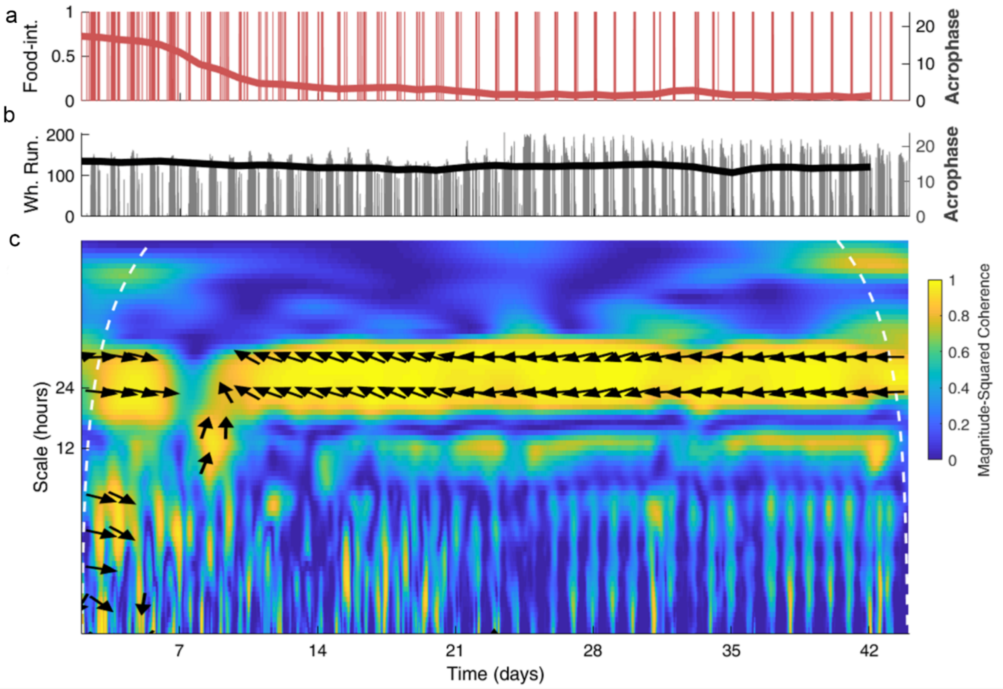

Supplementary Figure 7. Examples of the fifth step, wavelet coherence, of the 5-step wavelet approach applied to the food-intake and wheel running time series. Vertical bars represent the **a**) food-intake and **b**) wheel running time series of a representative mouse (F187) from the CR-day feeding paradigm (30% caloric restriction with 24h food access starting at the beginning of the day). In both panels, tick lines indicate the daily acrophase estimation, calculated as the peak values in the real part of the Morlet cwt coefficients at the corresponding 24h time scales (see also Supplementary Figure 4 g,h). **c**) Wavelet coherence values, with yellow regions representing higher and blue lower values of coefficients. Black arrows indicate the phase relationships between both time series at the 24h time scale. Note the change in direction of arrows after the disruption induced by the shift in feeding paradigm.

For each feeding paradigm treatment group, examples of acrophase estimated from the wavelet coefficients overlaid on actograms for food-intake (red) and wheel running (dark gray) are shown in the first and third column Supplementary Figure 8 (panels a, f, k, p, u and c, h, m, r, x, respectively) as well as the corresponding power estimates in the second and fourth column (panels b, g, l, q, v and d, i, n, s, y, respectively) and wavelet coherence between both behavioral time series in the fifth column (panels e, j, o, t, z). The color scheme represents the magnitude squared coherence. As expected, strong coherence (yellow) between behaviors, is observable at the 24 h scale over most of the experiment. At the 12 h scale, this yellow region can change over time and is associated with the feeding paradigm, disappearing in CR treatment groups altogether, consistent with the absence of rhythm in food-intake time series.

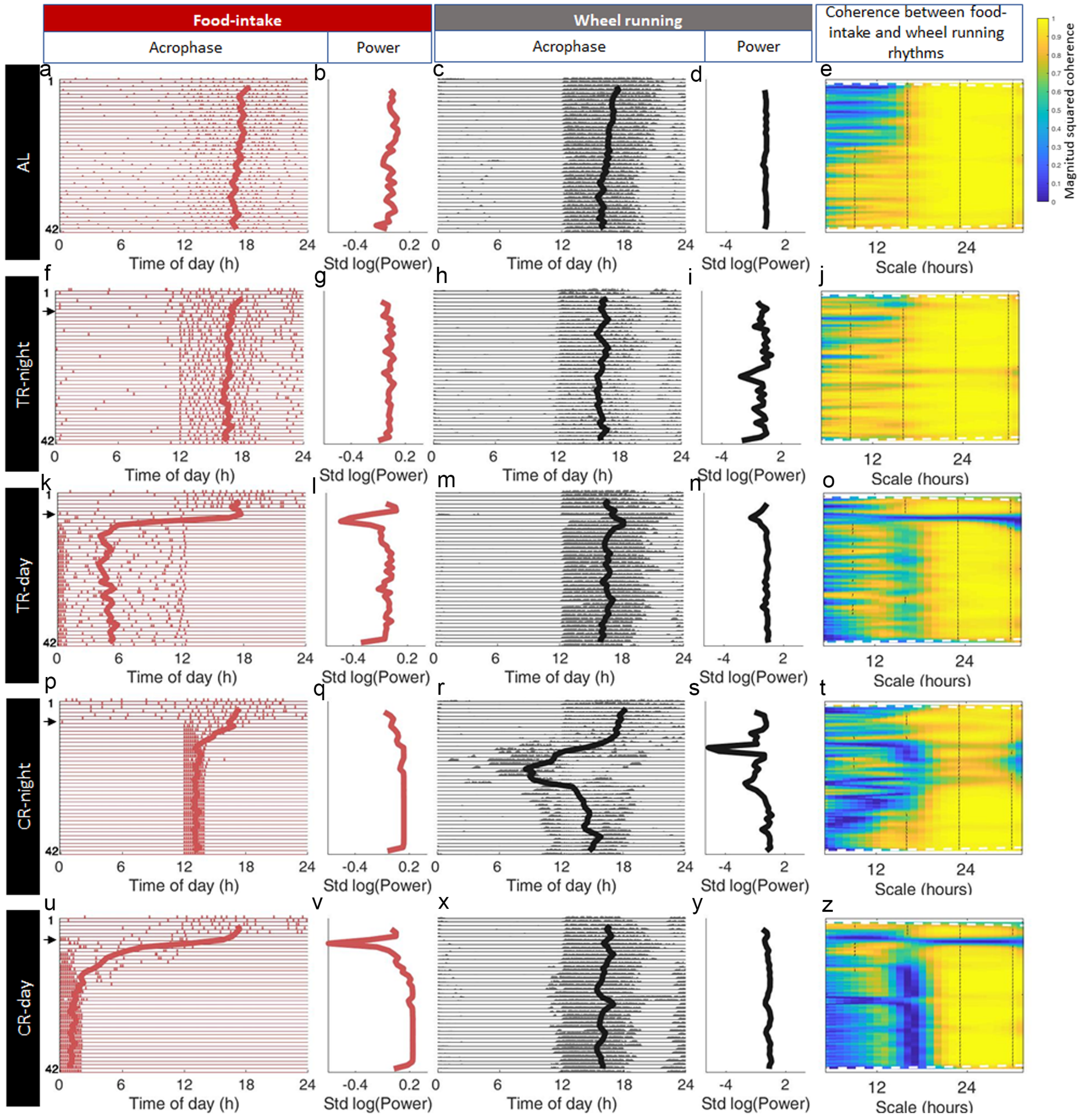

Supplementary Figure 8. Examples of modulation of values of acrophase, power and coherence estimates induced by feeding paradigm throughout testing in food-intake and wheel running time series. Each row shows the analysis of the food-intake (red) and wheel running (dark gray) time series of a representative mouse from each feeding paradigm as indicated by row header. Black arrow indications the transition to the novel feeding paradigm. (a,f,k,p,u) Food-intake and (c,h,m,r,x) wheel-running actograms are shown together with acrophase estimation (solid line). Acrophase was estimated as peak values in the real part of the Morlet cwt coefficients at the corresponding 24h time scales (Supplementary Figure 6). Power was estimated as the maximum squared modulus of the complex Synchrosqueezing coefficients at the 24±1h scale. Power values were transformed using logarithms and then standardized to the baseline value (i.e. mean values obtained between days 2-5 of experimentation). The last column shows wavelet coherence values, with yellow regions representing higher and blue lower values of coefficients. AL: *ad libitum*. TR-night and TR-day: 12h food access during the night or day, respectively. CR-night and CR-day: 30% caloric restriction with 24h food access starting at the beginning of the night or day, respectively.

Examples of phase relations between behaviors estimated with wavelet coherence analysis are represented with arrows in Supplementary Figure 8 in the fifth column (panels e, j, o, t, z) and Supplementary Figure 9 a-e for all treatment groups. Note that both coherence (color scheme) and phase (arrows in Supplementary Figure 9 a-e) remain relatively constant throughout the experiment in the AL and TR-night examples (Supplementary Figure 9 a, b). However, in the other three treatment groups disruption in coherence for scales around the 24h is observed as a change in coloration from yellow to blue (Supplementary Figure 9 c-e). Moreover, shift in the phase relationships between behavioral time series is clearly observed by the change in the angle of the arrow from approximately a 0º angle during the first week of experimentation (ad-libitum) to 180º angle after transition to the TR-day and CR-day feeding paradigms (Supplementary Figure 9 c,e). Quantification of the phase relationship of 24h-rhythms between behaviors during the last week of testing is shown in Supplementary Figure 9 f. It is evident that not only is there a phase advance in TR-day and CR-day, but also, to a lesser extent, a phase advance in TR-night.

To quantify the level of synchronization between animals in rhythm during the last week of testing the correlation coefficient was estimated (see methodology in Supplementary Figure 6) for both 24h and 12h time scales (Supplementary Figure 9 g-j). Significant effects of the feeding paradigm were observed. CR-night showed the highest level of correlation in 24h- food-intake rhythms (Supplementary Figures 9 g), while TR-d presented the highest values of correlation coefficients between conspecifics in 24h- wheel running rhythms and in the rhythm of both behaviors.

To assess the performance of our GaMoSEC 5-step wavelet approach to detect dynamical changes of rhythmic patterns in time series we analyzed the full 42-day behavioral time series. We also evaluated the changes in daily activity during the transition from *ad libitum* (AL) to a novel feeding paradigm (TR or CR), as well as the adaptation to the experimental conditions over time. Supplementary Figures 10 and 11 summarize the results

obtained by showing the achrophases and power of 24h- rhythms, respectively.

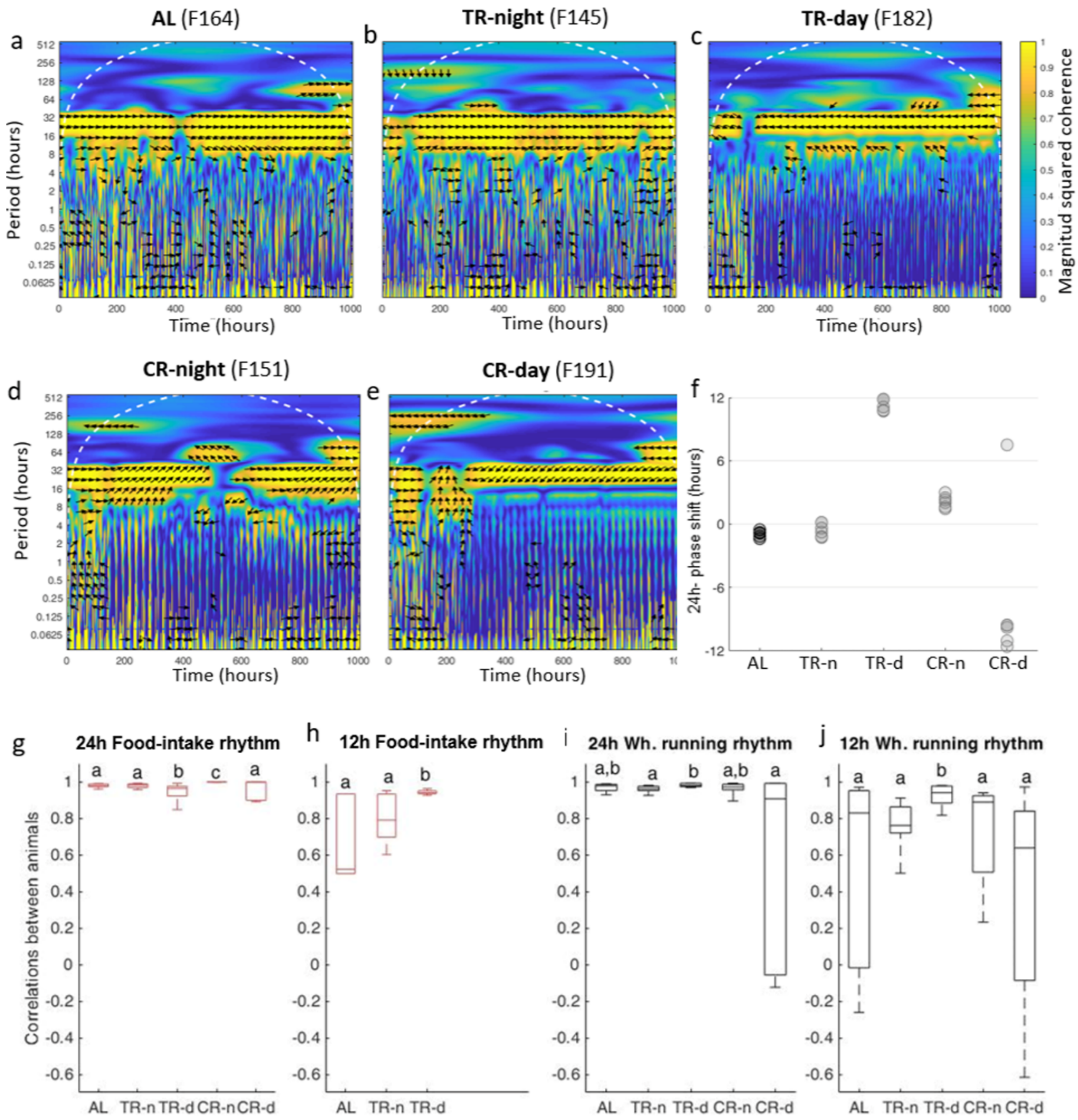

Supplementary Figure 9. Offset between peak food-intake and wheel running behavior, as well as level of synchronization between animals is dependent on imposed feeding paradigms. a-e) Example of coherence for the same time series shown in Supplementary Figure 8. The phase relationship is shown by the orientation of the arrows in the regions of high coherence (yellow). A rightward-pointing arrow indicates in-phase coherence between the two signals (Δφ = 0; phase shift of 0h), a leftward-pointing arrow indicates anti-phase coherence (Δφ = π; phase shift Period/2). The white dashed line shows the cone of influence where edge effects become significant. Note for the 24h scaling region in a, and b an arrow with a 0±45º angle (equivalent to a phase shift of 0 ± 3 hours) is observed in the yellow region of high coherence between behaviors over the entire testing time. However, in c, d and e the angle changes from a 0 ± 45º towards approximately 90 ± 45º, 180 ± 45º º and -90 ± 45º in the last 10 days of testing, indicating a phase shift of approximately 3,12, and -12h, respectively. **f**) Mean values of individual estimates of phase shift estimate are shown for the last 10 days of testing. **g-j**) Boxplots of correlation coefficients estimated between animals regarding 24h y 12h food-intake and wheel running rhythms. Box plots in panels g-j show the quartiles 1,2 and 3 and whisker depict the 5 and 95 percentiles. [a-d] Groups that do not share letters significantly differ P<0.05 (GLM, data transformed to ranks, and considering each individual

as a random factor).

Consistent with entrainment of 24h- rhythms with the LD cycle imposed, acrophases in both behaviors (running and feeding) under AL (Supplementary Figure 10 a and f) remains relatively constant over time (see also wavelet coherence analysis in Supplementary Figure 8). No phase changes were detected after the feeding paradigm switch from AL to temporal restriction during the night (TR-night) (Supplementary Figure 10 b,g). Conversely, acrophases of food-intake 24h-rhythms in mice subjected to a change from AL to TR-day paradigm (Supplementary Figure 10 c) advances immediately after the new paradigm is introduced (Supplementary Figure 10 k-m). This observation is consistent with the shift observed in the time required to consume 50% of total daily food-intake shown in Acosta-Rodriguez et al. 2017 [3]. The new acrophase stabilizes ~5 hours after food onset (Supplementary Figure 10 c). However, in TR-day the acrophases of wheel running remain unchanged (Supplementary Figure 10 h, n), consistent with Acosta-Rodriguez et al. 2017 [3].

Mice subjected to a change from AL to CR paradigm (both, CR-night and CR-day mice) showed acrophases advances in feeding behavior (Supplementary Figure 10 d, e, k), but, surprisingly, also in running wheel activity (Supplementary Figure 10 i, j, n). Shifts in feeding acrophases follow food onset regardless of when the onset occurs (day or night) and reflect the timing of the 2 h-temporal restriction of food-intake self-imposed by these mice [3]. Notably, the new phase relationship between food onset and feeding activity is precise (1 h for CR-night and 1h for CR-day). On the other hand, phase advances in wheel running are less pronounced and more variable, since there are individuals more sensitive than others to feeding changes (dot lines in Supplementary Figure 10 i and j). These results are consistent with the shift in running activity toward the daytime previously reported [3] and reflect a clear interference of CR on the wheel running phase establishment with regards to the LD cycle imposed.

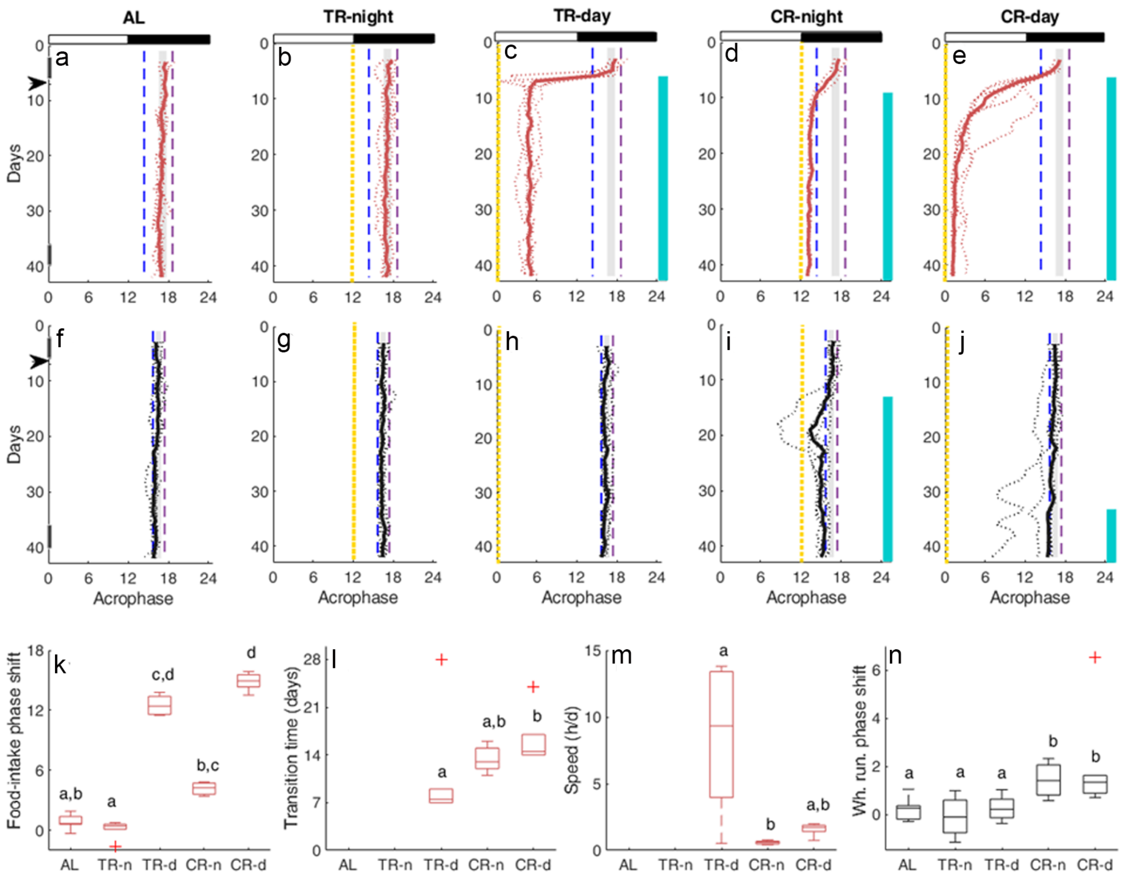

Supplementary Figure 10. The feeding paradigm has the potential to modulate the acrophase of both food-intake and wheel running 24 h - rhythm. a-e) food-intake and **f-j**) wheel running acrophases estimated using the wavelet method shown in Supplementary Figure 1 is plotted as a function of time for each feeding paradigm. **a,f**) *ad libitum*, **b,g**) TR-night, **c,h**) TR-day, **d,i**) CR-night, and **e,j**) CR-day. Bold red or black lines represent the median group values while respective dotted lines represent the values of each of the 6 individuals. Black arrow shows the moment of transition to the novel feeding paradigm. Acrophase was estimated as peak values in the real part of the Morlet cwt coefficients at the corresponding time scales (see graphical representation in Supplementary Figure 4), 24h scale. As a reference, percentiles estimated from the first week of *ad libitum* feeding were included. The gray area indicates the 25-50%, while the blue and purple dashed lines the 5% and 95% percentile, respectively. Right-side cyan bars mark days in which median acrophase values were lower than the 5% percentile. The yellow line is the time of day in which the feeding is initiated in each paradigm. Statistical comparison of paradigms using a Kruskal Wallis test of k) phase shift in acrophase of 24 h food-intake rhythm between the beginning and end of the experiment (H=26.68; P<0.0001, n=6), **l**) experimental day in which animals reached the phase shift (H=7.38; P=0.02), **n**) speed of transition: phase shift divided by the number of days needed to reach the phase shift (H=9.72; P= 0.008), and **m**) phase shift in acrophase of 24 h wheel running rhythm (H=16.28; P= 0.003). Box plots in panels k-n show the quartiles 1,2 and 3 and whisker depict the 5 and 95 percentiles. [a-d] Groups that do not share letters significantly differ P<0.05. Periods used for

estimations are indicated as dark gray bars on the x-axes on panels a and f.

Supplementary Figure 11 shows how the strength of 24 h - rhythms can be modulated by the feeding paradigm. This metric is related to the amplitude of the 24 h - rhythms. As expected, AL mice rhythms exhibit a consistent power throughout the whole experiment (Supplementary Figure 11 a, f). Nevertheless, for the other feeding paradigms groups we detected strengthening (magenta boxes) or weakening (cyan boxes) of the rhythms during different time periods along the experiment, which depend on the behavior observed (running or feeding) and feeding paradigm imposed. food-intake rhythms of TR-night mice (Supplementary Figure 11 b) show a tendency to strengthen the rhythmicity after the change in feeding paradigm, which is significant at specific days (19 and 30 after food paradigm change). A similar trend to strengthen rhythmicity is observed for the wheel running activity (Supplementary Figure 11 g) but followed by a significant and transient weakness of the rhythm (cyan box at day 23-24) before returning to initial strength levels.

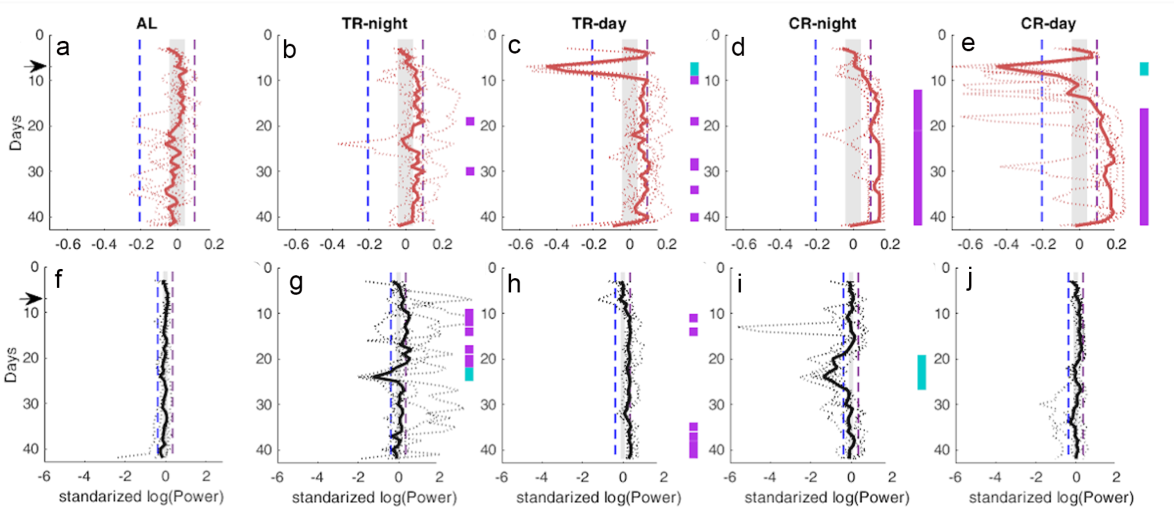

Supplementary Figure 11. The feeding paradigm has the potential to modulate the strength of 24 h - rhythms and introduces regions of weakness in wheel running 24 h - rhythm. Standardized values of the power of **a-e**) food-intake and **f-j**) wheel running 24 h rhythms as a function of time for each feeding paradigm as indicated by column headers. Bold red or black lines represent the median group values while respective dotted lines represent the values of each of the 6 individuals. Black arrow indicates transition to the novel feeding paradigm. Power was estimated as the maximum squared modulus of the complex Synchrosqueezing coefficients at the 24±1h scale. Power values were transformed using logarithm and then normalized to the baseline value (i.e. mean values obtained between days 2-5 of experimentation). As a reference, percentiles estimated from the first week of *ad libitum* feeding were included. Baseline percentiles are represented with horizontal markers, blue and purple dotted lines represent the 5% and 95% percentiles, and the gray box the 25% and 75% percentiles. Right-side cyan bars mark days in which median acrophase values were lower than the 5% percentile, indicating a temporal window of rhythm weakness. Right-side magenta bars indicate days strengthening of the 24 h - rhythm by the change in feeding paradigm.

For TR-day mice the strength in 24 h feeding rhythms abruptly decreases immediately after change in feeding paradigm is introduced, suggesting a short temporal "*window of rhythm weakness"* (Supplementary Figure 11 c). This window of rhythm weakness lasts 2 days after feeding paradigm transition, and then the power rapidly recovers and even strengthens transiently and intermittently (Supplementary Figure 11 c). The wheel running rhythm significantly strengthens after the transition, which is more evident by the end of the experiment (Supplementary Figure 11 h). Thus, transition from AL to TR-day affects differentially the strength 24 h - rhythms of behaviors, weakening the feeding and strengthening the running activity right after change, but by the end of the experiment both behavioral rhythms are strengthened.

Interestingly, the power of the food-intake rhythm in CR-night mice (Supplementary Figure 11 d) significantly and systematically increased over time, stabilizing 6 days after change in feeding paradigm. By contrast, wheel running (Supplementary Figure 11 i) does

not show a rhythm strength, instead a window of rhythm weakness is observed 13 to 19 days after tha feeding paradigm switch. This window of rhythm weakness is associated with the dramatic increase in daytime wheel-running activity occurring under CR paradigms (most pronounced in the CR-night fed mice) previously reported [2]. Noticeably during the same temporal window, the nocturnal activity transiently presents a phase advance (Supplementary Figure 10 i).

Lastly, we also detected a ***window of rhythm weakness*** in feeding rhythms for CR-day mice occurring immediately after the feeding paradigm change (Supplementary Figure 11 e), resembling that observed after change from AL to TR-day paradigm (Supplementary Figure 11 c). This window lasts 2 days but it exhibits a slower recovery of the strength over time (compare in Supplementary Figure 11 panels c and e). By day 17 of the experiment (10 days after transition), and thereafter, the rhythm significantly strengthened. The strength of wheel running rhythms seems not affected by this feeding paradigm (Supplementary Figure 11 j).

Summarizing, our approach provides new insights on the dynamics of phase transitions and strength of 24 h rhythms induced by different feeding protocols, complementing the previous observations by Acosta-Rodriguez (2017) [3]. The advantage of our methodologies is the ability to monitor daily changes in phase and strength of rhythmicity derived from the new feeding paradigms introduced, which act as dynamical perturbations on behaviors. Analyzing how these two parameters evolve throughout the experiment adds new layers of information regarding the underlying processes. It also allows us to characterize the subtleties of the interaction between two concomitant and/or conflicting external synchronizing signals (feeding paradigm and the LD cycle) affecting two major animal behaviors in mice.

In mammals, there is a network of circadian clocks, proposed to be hierarchically organized, with a ‘master clock’ located in the suprachiasmatic nucleus (SCN) of the

hypothalamus. The SCN tightly controls the sleep–wake cycle, hormonal rhythms and has a major role on the daily timing of food-intake [4] and is resistant to the synchronizing effects of food-intake timing, especially in presence of a LD cycle [5,6]. Consistently, we observed that the TR-night paradigm does not alter the phase of 24 h rhythms of either behavior. However, it does alter intermittently their strength. The strength of 24 h rhythmicity also was modulated by the TR-day paradigm. Moreover, all the behavioral perturbations introduced by the TR-day on feeding activity (phase advance with a concomitant window of rhythm weakness followed by significant strength of that rhythm) and wheel running (strengthen of the rhythm at the end of the experiment) are probably independent of synchronization of SCN by mealtime.

Beyond the SCN, there are other brain nuclei and peripheral tissues that contain weaker circadian clocks and can be phase-adjusted by feeding time [7,8], while humoral and visceral pathways convey metabolic status from periphery to the brain [9]. Some of the brain food entrainable clocks that participate in the circadian control of feeding, are reset by the peripheral metabolic signals coming from peripheral tissues and are related to the food clock. When access to food is restricted to the resting phase, the food clock drives an increase in the wheel running before the expected phase of food availability, producing a food-anticipatory activity, even in SCN-lesioned animals [10]. This food-anticipatory activity is faintly observed in wheel running activity of mice under TR-day, but increased under the CR paradigms [2]. Our analysis supports that caloric restriction, independently of starting at either the beginning of the night or the day, acts as a powerful interference for the phase locking to LD cycle of both behaviors, feeding and running. Specifically, they produced phase advances of 24 h activity rhythm. Consistently, it has been previously shown that timed calorie restriction in mice always leads (whatever the time of day at which limited food was given) to a phase advance of the SCN [11]. On the SCN, the CR paradigms shortens the endogenous period (tau) [12]; alter the expression of clock genes and neuropeptides

[13, 14, 15], and the expression of light-induced clock and clock-controlled protein [15]. Also, chronic CR paradigms affect LD-entrainment of SCN [16], likely by modulating phase response curves [14, 17].

Here we show that while both behaviors are affected by the CR paradigm, the perturbation on each one is different, and therefore we hypothesize that they occur via different mechanisms. Food-intake activity is more sensitive to CR, responding from the first days after introduction of this paradigm, while the wheel running response was delayed and weaker. We would like to highlight here the usefulness of our wavelet approach to detect the dynamical differences between the phase and the strength (power) of 24 h rhythmicity amplitude for each behavior, along the experiment. Moreover, the modulation by feeding paradigm of these two parameters seem also to follow independent mechanisms. In addition, we also detected differences in CR depending on the restriction start time: CR-night produces a stronger effect not only in the phase, but also in the strength of the 24 h wheel running rhythm as compared with CR-day. Indeed, concomitantly to the phase advance observed in the wheel running activity of mice under CR-night, there is a transient weakening of 24 h rhythmicity (called window of rhythm weakness), overlapped with the food-anticipatory activity previously reported [3]. The strength of the 24 h wheel running rhythm restores after this window of rhythm weakness, whereas the phase remains advanced until the end of the experiment, confirming different modulatory pathways. Conversely, the CR-day paradigm does not change the rhythm strength of wheel running activity until the last days of the experiment, similarly to the food-anticipatory activity consolidation dynamics [3]. Hence, the temporal onset of food-intake (and therefore the timing of the self-imposed 2-h time restriction induced under the CR paradigm and the food-anticipatory activity associated), seems to be critical for the perturbations on wheel running activity, more than the CR itself.

Interestingly, both TR-day and CR-day show windows of rhythm weakness in food-intake time series, immediately after the food paradigm changed. This phenomenon does

not emerge under the CR-night paradigm, even when a phase advance in food-intake activity is observed as well. Thus, the window of rhythm weakness in feeding is consistent with a transitional state in mice, changing its feeding activity to the restime, with certain independence of the caloric restriction itself. Further inspection of the full experimental period also shows that the evolution towards the expected phase advance in feeding is dependent on the feeding paradigm. Not only changes in acrophase, but also power, are slower in the CR paradigm than TR-day. However, once the transition occurs, the resulting dynamics is more robust as indicated by a sustained higher value of power in the CR groups (Supplementary Figure 11d,e) as compared to TR-day (Supplementary Figure 11c) . The fact that different kinetics of phase shifts, final phase relationships and changes in the 24 h rhythm strength are observed in the behaviors under CR as compared to TR and AL are indicative of different processes underlying them and further studies are needed for elucidate the mechanisms or regulation pathways implicated under each LD cycle/ food paradigm interaction.

Supplementary Note 4: Scaling in behavioral time series estimation

DFA is one of the most commonly used methods to quantify the scale-free nature of physiological time series and their alteration in disease [18-20]. Supplementary Figure 12 shows two example time series (Supplementary Figure 12 a,b) in which DFA is performed. Supplementary Figure 12a shows a typical Cantor set, that has long-range correlations, while Supplementary Figure 12 b shows data following a random uniform distribution, with the range of values varying either between 0 and 1, or between 0 and 0.2, reflecting a circadian-like patter. Raw data of these two artificially constructed time series is provided in Supplementary Data 1.

Detrended Fluctuation Analysis (DFA) consists of five steps. In the first one, the cumulative $\{Y_t\}$ time serie is estimated from the raw time series data $\{X_t\}$, for each time point t ϵ {1,...N}:

$$Y_t = \sum_{j=1}^{t} X_j \qquad (1)$$

This integrated time series {Yt} is divided into [N/n] non overlapping window, each containing n observations (Supplementary Figure 12 b, c, colored dotted lines). In the third step, for each block, a least square line is fitted to the data, which represents the local trend of the block (Supplementary Figure 12 b, c, colored lines). In the fourth step, the time series {Yt} is detrended by computing

$$Z_t = Y_t - Y_t^n \qquad (2)$$

where Y_t^n denotes the adjusted fit on each block. Finally, in the fifth step, for each nϵ{2m+2,...,N/4}, the

$$F(n) = \sqrt{\frac{1}{n}\sum_{t=1}^{M}(Y_t - Y_t^n)^2} = \sqrt{\frac{1}{n}\sum_{t=1}^{M} Z_t^2} \qquad (3)$$

where M is the maximum multiple of n, smaller or equal to N, i.e. M=[N/n]. Notice that F(n) will increase with the window size (scale, n). A linear relationship on a log-log scale indicates the presence of power law scaling (Supplementary Figure 12 e, f):

$$F(n) = \varphi n^{\alpha} \qquad (4)$$

Under such condition the fluctuations can be characterized by a scaling exponent α, which is the slope line when regressing log(Fluctuation) on log(Scale) as shown in Supplementary Figure 12 e, f. Note that the difference between the two example time series are reflected in the estimated

When using DFA, long- term memory should not be a priori assumed; therefore, to reliably infer power-law scaling of the fluctuation function, a straight line in the log-log plot has to be established [21]. Since a straight line is tantamount to a constant slope, the local slopes for a range of window sizes of log Fluctuation (F(n)) as a function of window size (*n*) have to be evaluated for constancy in an adequate range [21, 22]. First, a straight line to log F (n) vs. log *n* within a small window is fitted. In the examples shown in Supplementary Figure 13 a and d, ten consecutive points (blue filled circles) in the double log plot of fluctuation *vs.* window size are fitted with a line, and the local slope (Supplementary Figure 13 b, e, green asterisk), R^2 (Supplementary Figure 13 c, f, green asterisk) and sum of squared residuals are estimated (not shown). Since the value of the local slope, R^2 of the linear fit and the sum of squared residuals depend both on the location and number of data points utilized in the fitting procedure, this fitting process was repeated by siding the fitting window consecutively and estimating parameters. Black and blue dots in Supplementary Figure 13 represent the values estimated using 6 or 10 data points, respectively.

The appropriate scaling range was determined as the region for which all animals

showed stable values of local slope, maximum coefficient of variation and minimum sum of squared residuals [23, 24]. For example, for α_1, this analysis showed that the use of 16 data points from window sizes log (~10min) and log (~75min) was optimal for α_1 estimation in all animals (indicated in cyan in Supplementary Figure 13).

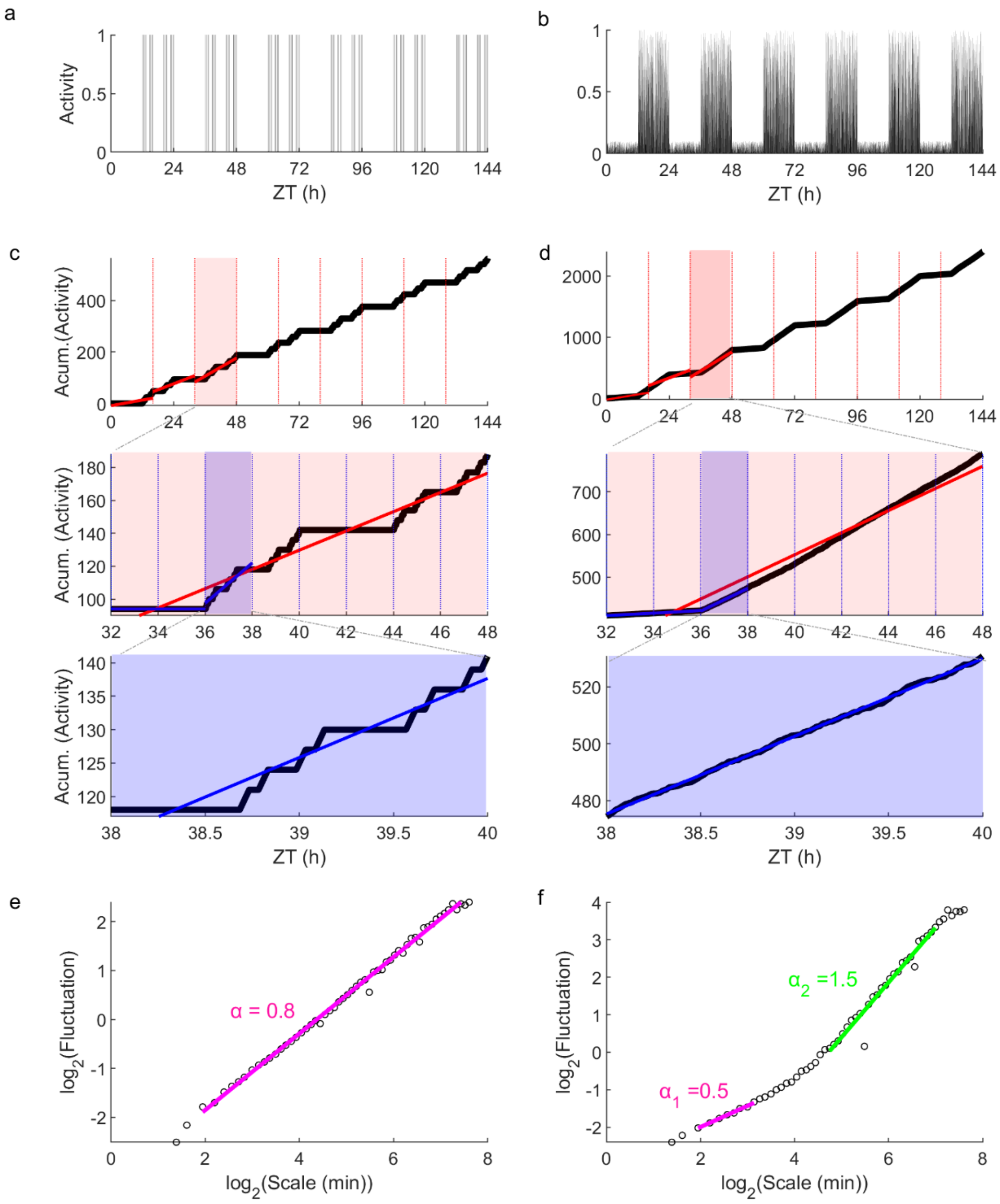

Supplementary Figure 12. Example of analysis of two artificially constructed activity time series with DFA. a, c, e) Left column represents shows the time series and analysis of a fractal cantor set with long-range

correlations. **b, d, f**) Right column shows the time series and analysis of random noise with a uniform distribution with amplitudes that vary periodically according to a circadian structure. **a, b**) Raw time series. **c, d**) Accumulative activity estimated from the time series shown above in panels a and b, respectively. In DFA, acumulative time series are divided into windows (color dotted lines) and each window fit with a polynomial function as represented in solid lines in the first three windows. Zooming in shows that by using smaller windows scales, fits better can copy the data. For each time window size (scale) the fluctuation can be estimated (see formula in text). **e, f**) Log-log plots of fluctuation as a function of the window size (scale). The slope of the linear relationship between them is the self-similarity parameter, α.

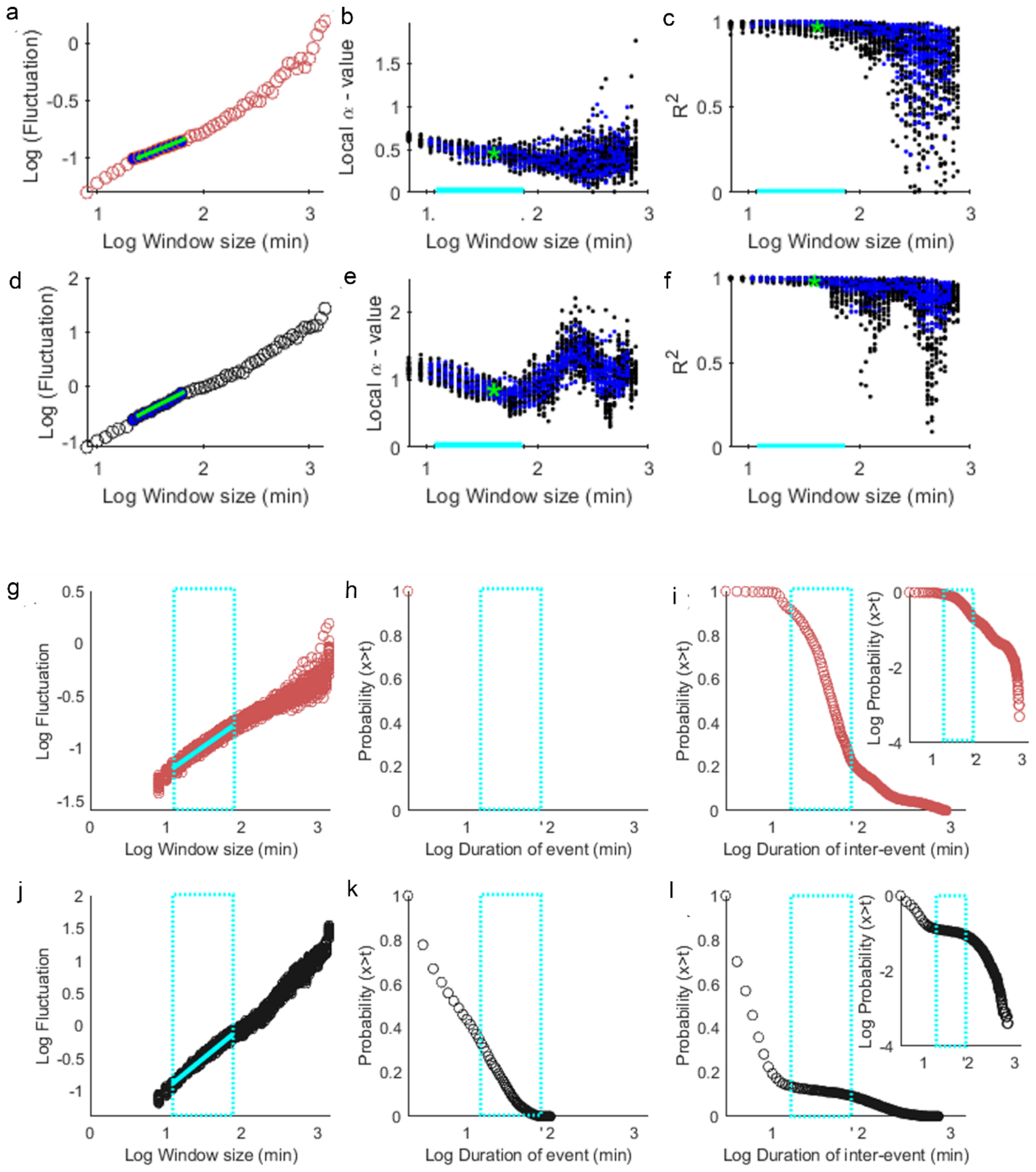

Supplementary Figure 13: Example showing the criteria utilized for selecting the optimal region for α-estimation. Third order DFA was performed on the same a-c) food intake and d-f) wheel running time series analyzed in Fig.1. a,d) Local α_1-values were estimated from the slope of the linear fit (green line) using a subset

of data points (blue filled circles), then the R^2 of the fit was calculated.. This process is then repeated over the entire data set for all possible length of data points and window size values. b, e) Local slopes, and c, f) R^2 of the corresponding linear fits estimated for different window sizes 6 (black dots) and 10 data points (blue dots). The corresponding local slope and R^2 estimated in panels a and d are marked as green asterisks. Note that bias in α estimation for 10 data points, shows greatest variability (b, e), and lowest R^2 (c, f), for large window sizes. Complete analysis showed that the use of 16 data points from window sizes log(~10 min) and log(~100 min) was optimal for α_1 estimation in all animals (indicated in cyan on x-axis). g-l) Comparison between scaling region of Detrended Fluctuation Analysis used for α_1, and cumulative probability distributions of the duration of food intake and wheel running events and inter-events, *ad libitum*. g,j) Same plots as Figure 1c (main text), showing Detrended Fluctuation Analysis of food intake (red) and wheel running (dark gray) time series of all 30 animals. The linear region determined in panels a-e are indicated by the cyan rectangle. h,k) Probability distribution of food intake and wheel running events. Events are defined as a continued period of time that the animal performs a given behavior, considering the sampling interval of 1min). Due to the experimental setup that induces a minimum time lag of 10min between pellets, a feeding event can only have a value of 1min. i,l) Probability distribution of food intake and wheel running inter-events (i.e. time period between events). Insets show the same plot at double logarithmic scales. Note that in the DFA scaling region (cyan box) linearity is observed in both event and inter-event distributions (panels i,k,l).

As shown in Figure 1 panels i and j in the main text, *ad libitum* (first week of testing) behavioral time series can present multifractality, thus different processes characterize each time scale. This is also reflected in the probability distributions of behavioral events and inter-event durations that also present non-homogeneous distributions (Supplementary Figure 13 h,k) with changes in dynamics around 100 min. Given the feeding system used, feeding are discrete events (Supplementary Figure 13 h) that are separate in time by at least a 10 min period (Supplementary Figure 13 h). Afterwards, the cumulative probability of the duration of inter-events decays differently before (cyan box in Supplementary Figure 13 i) and after 100 min (Supplementary Figure 13 i). In wheel-running events, shows an exponential decay with events lasting predominately less than 100 min, (i.e. linearity in the semi-log plot shown in Supplementary Figure 13 k). For inter-events a pronounced change in slope is observed after the 100 min (i.e. see the double-log plot inset in Supplementary Figure 13 l).

This distinct dynamics observed below and above 100 min for event and inter-event cumulative probability distributions is also evident in mice under each of the different feeding paradigms given that the dynamics at the short and long time scales are modulated

differentially (Figure 3 main text). For example, the cumulative food-intake time series used as the starting point of DFA, in CR is step-like, since mice in the CR treatment groups only eat during a short time interval after food is presented (light red in Supplementary Figure 14 a) contrary to the *ad libitum* group (dark red Supplementary Figure 14 a). This is reflected by α_1-values below 0.5, indicating anti-correlation for short time scales, and α_2-values above 1 for longer time scales in all animals (Supplementary Figure 14 f,g). For wheel running, the cumulative time series presents fluctuations at small time scales (inset in Supplementary Figure 14 b) while at large time scales it appears smoother and linear. This change in pattern is as reflected as the increase in the slope in the double log plot after 100 min in all groups (Supplementary Figure 14 h). These two scaling regions continue to be evident in the cumulative probability distributions of events and inter-events in both behaviors in all treatment groups (Supplementary Figure 15).

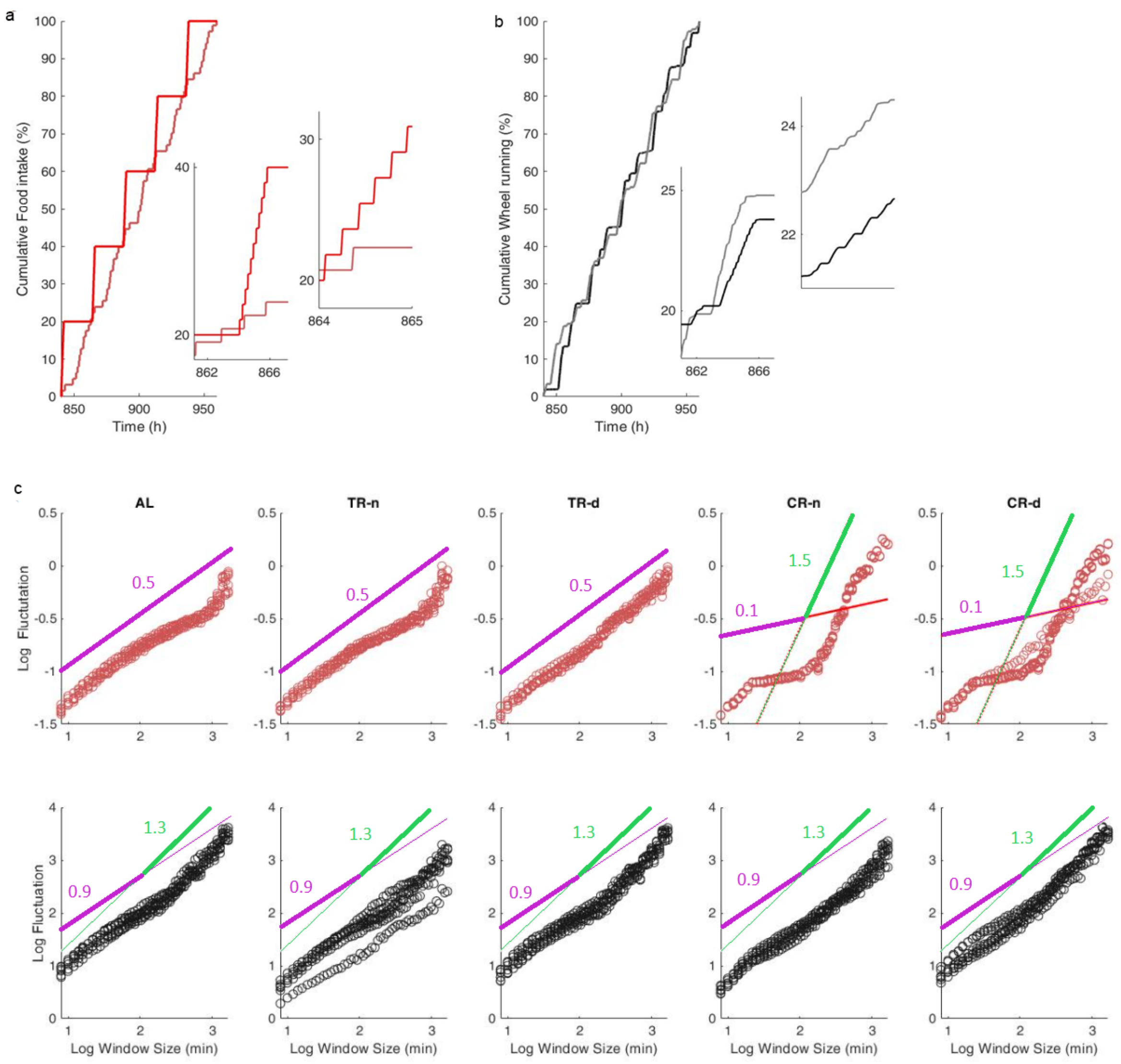

Supplementary Figure 14. Self-similarity and auto-correlation properties of behavioral dynamics under different feeding paradigms. **a**) Comparison between cumulative food-intake time series at progressively smaller scales of mice fed *ad libitum* (AL, dark red) and under caloric restriction (CR, light red) the last 5-days experimental period. Note the larger step-like pattern for CR, with less details compared to AL. **b**) Similar overall pattern is observed for both examples, AL (black) and CR (gray). **c**) Each panel represents DFA performed on all mice from each treatment group for food-intake (first row) and wheel running (second row). Purple and green lines with slopes of either 0.1, 0.5, 1.3 or 1.5 were added for visual reference. Actual mean group values are presented in Figure 3 c and d.

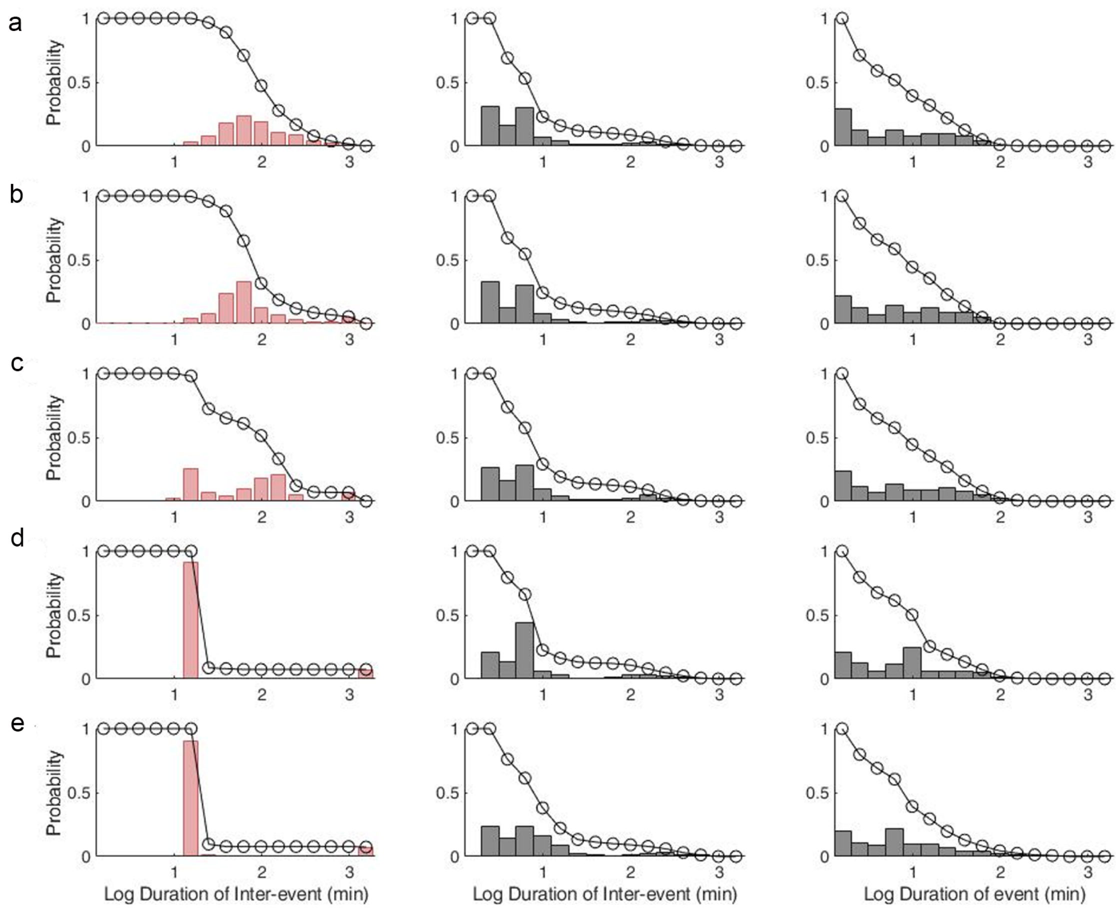

Supplementary Figure 15. Comparison between treatments in regard to the probability distribution and the cumulative probability distribution of durations behavioral inter-event and event durations in the final 5-days of testing. Bars represent probability distribution of food intake and wheel running inter-events (first and second columns, respectively) and wheel running events (third column) duration while the open circles and lines indicate the cumulative probability, i.e. the probability of an inter-event or event of having a duration equal or above a given duration. Events are defined as a continued period of time that the animal performs a given behavior, considering the sampling interval of 1 min. Due to the experimental setup that induces a minimum time lag of 10min between pellets, a feeding event can only have a value of 1 min, thus are not shown. Inter-events are defined as the time period between events. For each panel all inter-events and records for the animals in each treatment group were analyzed (**a**) AL: ad libitum. (**b**) TR-night: 12h food access during the night. (**c**) TR-day: 12h food access during the day. (**d**) CR-night: 30% caloric restriction with 24h food access starting at the beginning of the night. (**e**) CR-day: 30% caloric restriction with 24h food access starting at the beginning of the day. In this treatment animal F192 was excluded from analysis given the distinct pattern that it shows in comparison to the rest of the animals in its group as shown in all other analyses. Note that both CR groups present a remarkable shift towards durations between 10-20 min and long fasting periods. In regard to wheel-running, most inter-event lasted less or equal to 10min, while almost all events lasted less than 100 min, independently of experimental treatment.

Supplementary Note 5: Genetic differences in timing of food consumption seems not related to inter-individual differences in the expression of 12h-rhythms.

Although most mice have similar total daily food-intake and consolidate it during the night, others systematically consume 25% of food during the day [3]. Authors suggested that this intrinsic, genetic based, individual variability may be associated with the substantial variation in body weight among C57BL/6J individuals [3].

Given that 12h-rhythms could potentially be induced by a phase misalignment between the daily activities, we sought to analyze whether the individual (genetic background associated) differences in the timing of food consumption are related to the presence/intermittence of 12h-rhythms under the AL paradigm. Supplementary Figure 16 shows stacked histogram plots displaying the food-intake (a and c) or the wheel running activity (b and e) during the day or the night along the whole experiment for two individual mice (mice 166 and mice 167, see also Figure 2, main text) under AL paradigm. We included a vertical bar indicating the days in which we detect 12h-rhythms for each behavior. There is no apparent correlation between an increased food consumption during daytime and the presence of 12h-rhythms in the time series. This observation supports the hypothesis that the individual differences observed in the 12h-rhythms expression are not directly linked to their genetic background, previously associated with differences in the timing of food consumption.

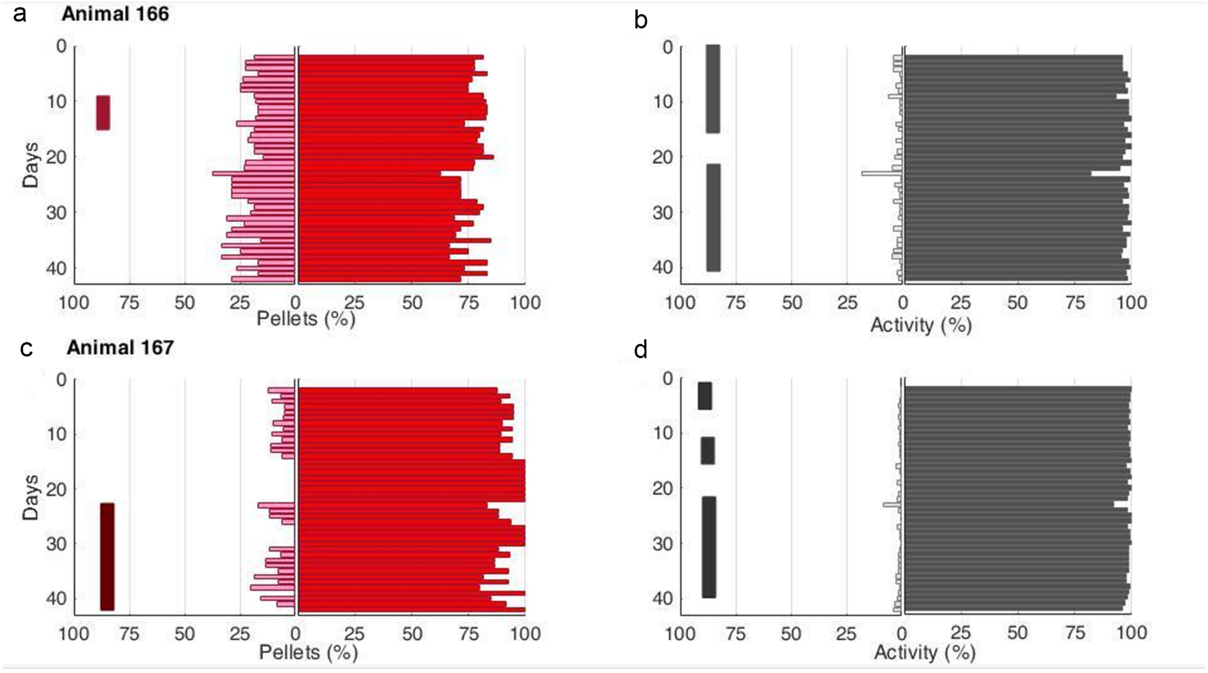

Supplementary Figure 16. Intermittent expression of food-intake and wheel running 12h rhythms are not directly associated with individual variation in food consumption during the day. Two examples of feed consumption and activity patterns (as shown in Supplementary Figure 3 in Acosta-Rodriguez et al (2017)) in relation to the presence or absence of 12h rhythm (vertical bar to the left of each panel, as in Figure 4, main text). Panels correspond to animal 166 (a, b) and 167 (c,d), respectively, from the AL group**.** a, c) A stacked histogram plot displaying the food-intake during the day or the night along the 43 experimental days for each individual mouse. Pink bars extended from the center to the left represent the daytime activity, whereas night activity recordings are plotted as red bars extended to the right. b, d) a stacked histogram plot showing the wheel-running activity distributed during the day or the night along the 43 experimental days for each individual mouse. Daytime activity is plotted as light gray bars extended from the center to the left. On the contrary, night activity recordings are plotted as dark gray bars extended to the right.

Supplementary Note 6: Acrophase and power analysis of 12h-rhythm under different feeding paradigms.

To explore the modulation of 12h-rhythms by feeding paradigm and its relationship with LD cycles, we estimate, when possible, the acrophase (Supplementary Figure 17) and the power (Supplementary Figure 18) of 12h-rhythms in running and food-intake activity. Data from individuals under each specific paradigm group, along the whole length of the experiment is shown, as well as the median acrophases of 12h-rhythms present during the first week of testing, in which all the mice were under AL for reference.

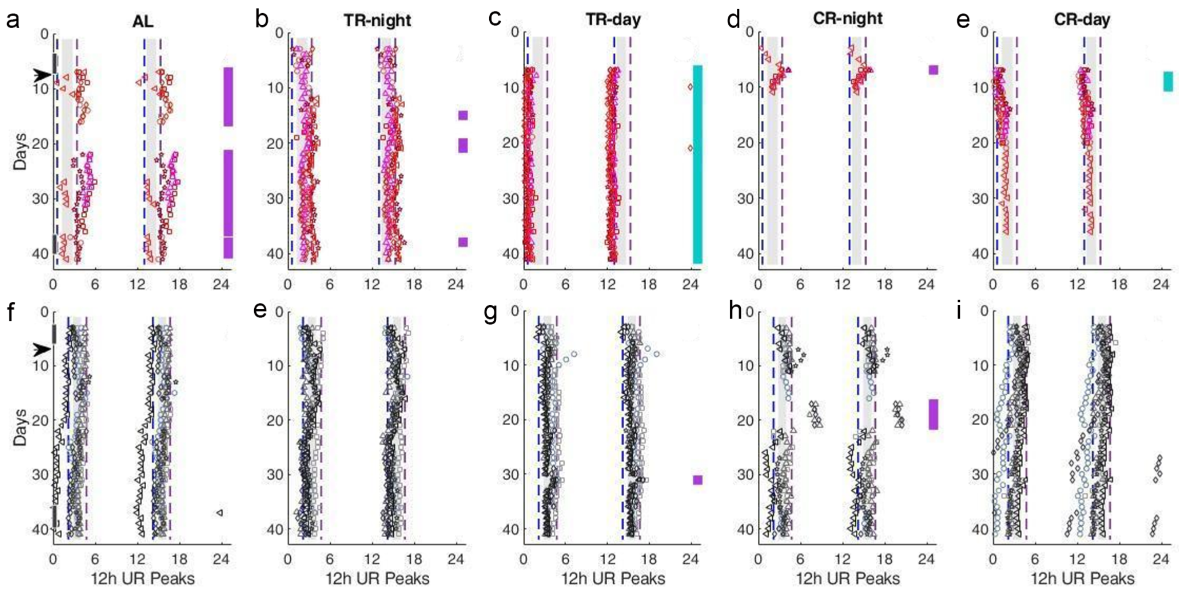

Supplementary Figure 17. The feeding paradigm has the potential to modulate the phase of both food-intake and wheel running 12h ultradian rhythms. The time of day of the two peaks in a-e) food-intake and f-j) wheel running of the 12h rhythm was estimated in each period in which these rhythms were detected (see Figure 4) using the 5-step wavelet method. Each symbol represents a specific animal. Black arrow shows the moment of transition to the novel feeding paradigm. a,f) *ad libitum*, b,g) TR-night, c,h) TR-day, d,i) CR-night, and e,j) CR-day. Acrophase was estimated as peak values in the real part of the Morlet cwt coefficients at the corresponding time scales 12h scale. As a reference, percentiles estimated from the first week of *ad libitum* feeding were included (n=30). The gray area indicates the 25-50%, while the blue and purple dashed lines the 5% and 95% percentile, respectively. Right-side cyan bars mark days in which median phase values were lower than the 5% percentile. Statistical comparison of paradigms using a Kruskal Wallis test of the values of the first peak in the rhythm in k) food-intake and l) wheel running during the last 5-days of experimentation. * Group significantly

differed from AL and TR-night ($P<0.05$).

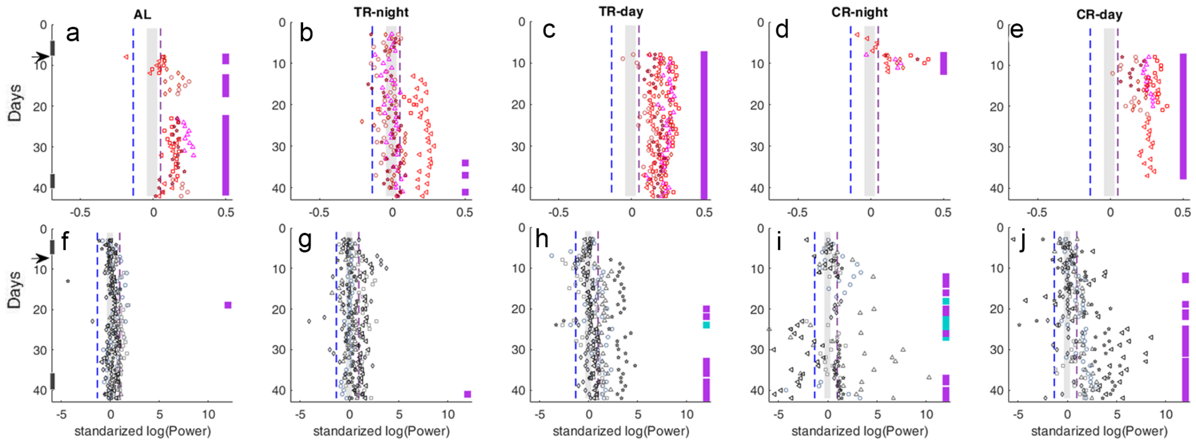

Supplementary Figure 18. The feeding paradigm has the potential to strengthen food-intake and wheel running 12h ultradian rhythms. Standardized values of the power of a-e) food-intake and f-j) wheel running of the 12h rhythms were estimated in each period in which these rhythms were detected (see Figure 4) using the 5-step wavelet method. Each symbol represents a specific animal. Black arrow shows the moment of transition to the novel feeding paradigm indicated by the header. Acrophase was estimated as peak values in the real part of the Morlet cwt coefficients at the corresponding 12h time scale. As a reference, percentiles estimated from the first week of *ad libitum* feeding were included (n=30). The gray area indicates the 25-50%, while the blue and purple dashed lines the 5% and 95% percentile, respectively. Right-side cyan bars mark days in which median phase values were lower than the 5% percentile. Power values were transformed using logarithm and then normalized to the baseline value (i.e. mean values obtained between days 2-5 of experimentation). Statistical comparison of paradigms using a Kruskal Wallis test of the values of the first peak in the rhythm in k) food-intake and l) wheel running during the last 5-days of experimentation. * Group significantly differed from AL and TR-night (P<0.05).

In the TR paradigms, especially during the day (TR-day), present low inter-treatment variability is observed. This is consistent with the strong positive correlations between animals (mostly above 0.7, Supplementary Figure 9 g-j) shown previously, with TR-day showing a significantly (P<0.01) higher level of synchronization in comparison to AL (Supplementary Figure 9 h, j). These suggest that TR paradigms cooperate in reducing the inter-individual variability in the phase of 12h-rhythms, when detected.

For the food-intake time series, the introduction of the TR-day paradigm produces a significant phase advance of the 12h-rhythms as compared

with the AL paradigm throughout the experiment. Note that the CR-day paradigm also introduces a significant phase advance in food-intake time series but only for a few days immediately after feeding paradigm change. However, afterwards, as shown in Figure 2 of main text, CR abolishes the presence of 12h-URs.

Acrophases of 12h-rhythms detected in wheel running time series are insensitive to feeding paradigm changes. However, note that for the CR-night paradigm a transient breakout of phase estimation in the 12h-rhythm of wheel running is observed between the 15th and 32nd day post feeding paradigm change (violet box). This is consistent with the discontinuities in 12h-rhythms expression observed in every mouse of that group (see Figure 2 i). Afterwards, achrophases recover their previous phase.

We also estimate the strength of 12h-rhythm in wheel running and food-intake activity by power analysis (Supplementary Figure 18). We used as reference the median power of 12h-rhythms present during the first week of testing, in which all the mice were under AL conditions. Results of the AL group reveal that 12h-rhythms for feeding activity, when present, increase in strength with the length of the experiment (Supplementary Figure 18 a, note that the increase is in logarithmic scale), which may suggest consolidation of these rhythms over time. Conversely, the TR-night paradigm seems to maintain the relative strength of feeding 12h-rhythms throughout the experiment (Supplementary Figure 18 b). Hence, suggests that the TR-night counteracts the increase in strength observed under AL. This effect is not observed for the 12h-rhythms of wheel running activity under AL or TR-night paradigms. Surprisingly, the TR-day significantly increases the power of 12h-rhythm in both food-intake and wheel running activities (Supplementary Figure 18 c, h).

In TR-day an immediate increase in power is observed in feeding 12h-rhythms (Supplementary Figure 18 c). Surprisingly, in this group an increase in the strength in wheel running rhythms is slowly achieved by the end of the experiment (Supplementary Figure 18 h). As previously shown, the CR paradigms tend to abolish or decrease the expression of rhythms in feeding activity, and therefore there is very little data for exhaustive analysis (Supplementary Figure 18 d,e). Nevertheless, for the wheel running activity, CR paradigms (both, CR-night and CR-day) increase the dispersion of strength among individuals during the experiment, but only CR-day significatively strengthens these rhythms (Supplementary Figure 18 i,j).

By evaluating the change in presence/absence of rhythms over time, together with the corresponding modulation of achrophases and strength we show the sensitivity of these rhythms to external cues.

Further experiments are required for elucidating whether food or lighting conditions are functional zeitgebers affecting 12h-rhythms and how the interaction between these external signals impact on these dynamical patterns.

Supplementary Note 7: Modulation of short- and long-range autocorrelation properties induced by the feeding paradigm over time.

We explore the potential for specific feeding paradigms to modulate the short- and long-range autocorrelation properties observed in food-intake and wheel running (Supplementary Figure 17), respectively, that are apparent at the short time scales. As expected, mice under the AL paradigm exhibit α_1-values that remained relatively constant throughout the experiment, in both feeding and running activities (Supplementary Figure 19 a, f). Moreover, these two behaviors continue to show throughout the experiment contrasting dynamics at time scales < 100 min. At this time scale food-intake time series show random or short-range correlations, while long-range correlations were detected in wheel-running activity. These dynamical properties at short time scales were also observed in mice exposed to the TR-night (Supplementary Figure 19 b, g) throughout the experiment. Contrarily, TR-day, CR-night and CR-day mice present a decrease in α_1-value in food-intake in the days following the switch from AL to the feeding paradigm (Supplementary Figure 19 c-e). As stated in the previous supplementary note, these α1-values between 0 and 0.5 indicate a change in the properties of the dynamics from random or short-range correlations to anti-correlation. Anti-correlation in this context can be associated with the periods of feeding being followed by prolonged non-feeding episodes. Moreover, as shown in the main text, by the end of the experimental period these three groups showed significantly lower α_1-value in comparison to the AL group ($P<0.05$, Figure 3 c).

In wheel-running time series, these mice showed localized decreases in the α_1-value at specific time points, comparable to the alterations in dynamics observed at the 24h- and 12h- rhythms. In both TR-day and CR-day mice these decreases are noticeable on the days following the switch in paradigm, namely the 8th and 12th day of experimentation, while for CR-night this is observed by the 24th day (Supplementary Figure 19 h-j). By the end of the experiment α-values showed a tendency (P<0.1, Figure 3 d) to be lower in TR-day and CR-night and were significantly lower in CR-day (P<0.05, Figure 3 d) in comparison to AL mice. Thus, further highlighting the impact of the feeding paradigm not only on the temporal dynamics of food-intake but also on wheel running.

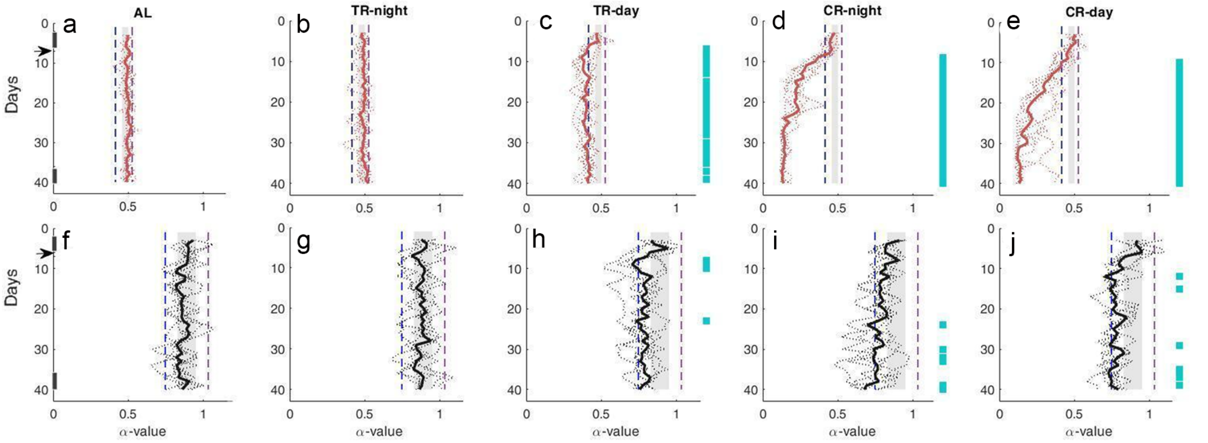

Supplementary Figure 19. The feeding paradigm modulates auto-correlation (fractal) properties of short-scale wheel running dynamics. α_1-values of a-e) food-intake and f-j) wheel running 24 h - rhythms estimated with Detrended Fluctuation Analysis are plotted as a function of time for each feeding paradigm. Bold red or black lines represent the median group values while respective dotted lines represent the values of each of the 6 individuals. Black arrow shows the moment of transition to the novel feeding paradigm. (a,f) AL: ad libitum. (b,g) TR-night: 12h food access during the night. (c,h) TR-day: 12h food access during the day. (d,i) CR-night: 30% caloric restriction with 24h food access starting at the beginning of the night. (e,j) CR-day: 30% caloric restriction with 24h food access starting at the beginning of the day. As a reference, percentiles estimated from the first week of *ad libitum* feeding were included. Baseline percentiles are represented with horizontal markers, blue and purple dotted lines represent the 5% and 95% percentiles, and the gray box the 25% and 75% percentiles. Right-side cyan bars mark days in which median acrophase values were lower than the 5% percentile.

Supplementary Note 8: Correlation matrix and scatter plots between variables

Supplementary Figure 20 shows the relationship between food-intake and wheel running behaviors regarding circadian acrophase (Supplementary Figure 20a), persistence in 12h rhythms (Supplementary Figure 20b), α_1-values (Supplementary Figure 20c), and α_2-values (Supplementary Figure 20d). As expected (see Supplementary Figure 10K and [3]), food-intake acrophase of daily rhythms (Supplementary Figure 20a) separated treatment groups, while in themselves the other variables do not fully separate treatment groups. Compare to Principal component analysis in Figure 4b (main text), when variables are analyzed together, showing maximum separation between groups.

Supplementary Figure 21 shows the correlation matrix between dynamical behavioral variables and physiological variables obtained by the end of the experiment. Significant correlations ($P<0.05$) are shown with a light grey background. Note that none of the coefficient of determination was larger than 0.58. Therefore, a multivariate PCA approach, as illustrated in Figure 4, proves more useful for understanding data variability associated with feeding paradigms. Autovectors and correlations between variables with the first two principal component axes of each panel of Figure 4 (main text) are shown in Supplementary Tables 1-8, respectively.

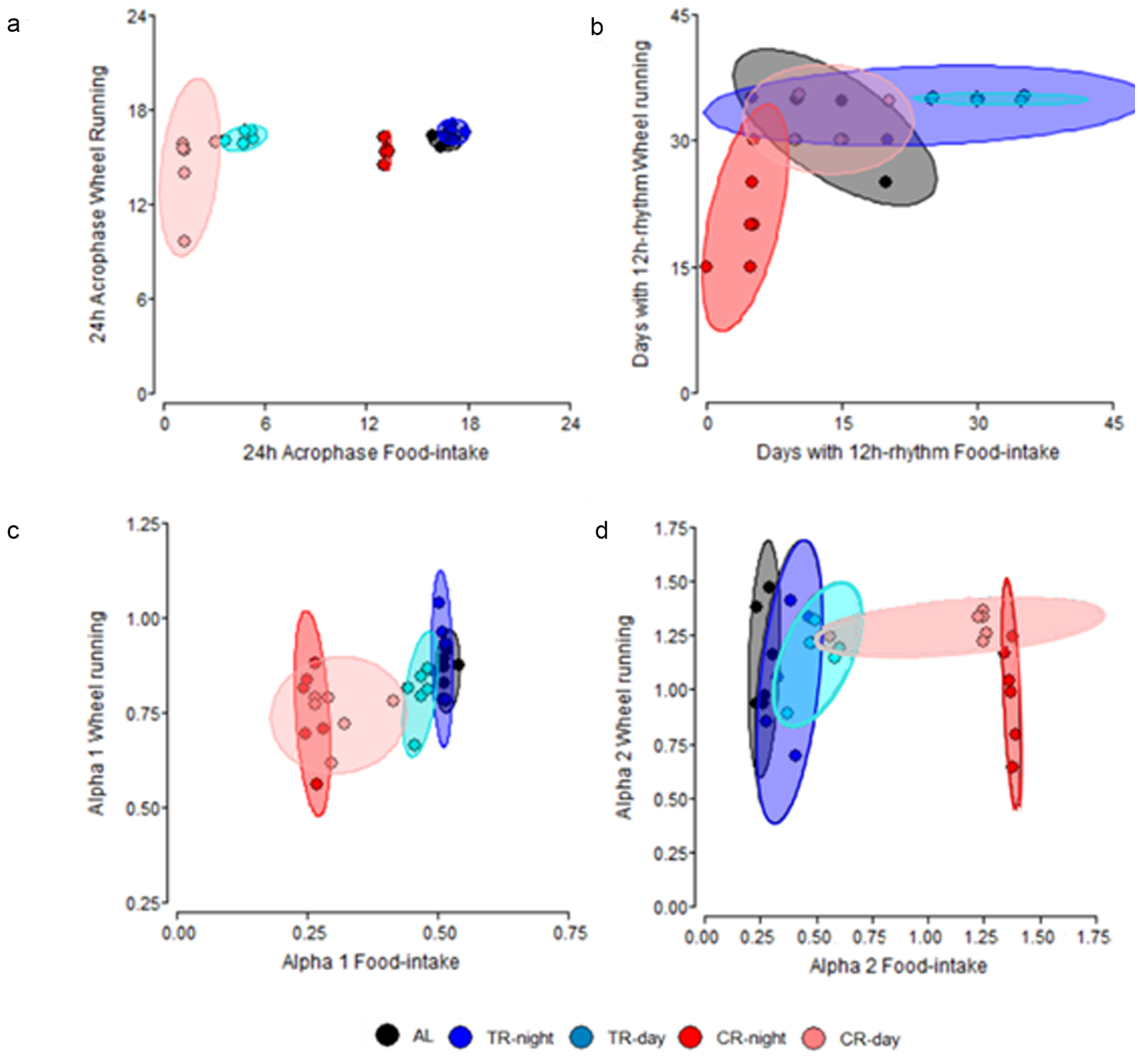

Supplementary Figure 20. Scatter plots of mice under different feeding paradigms. Each circle represents an individual, with the color denoting the treatment as indicated in the legend key. a) Food-intake and Wheel running acrophase associated with the circadian rhythm is the same as data shown in Figure 2. b) The number of days in which 12h URs were observed in each behavior is the same as shown in Figure 2Q and L, as with c-d) α-values in Figure 3c and d.

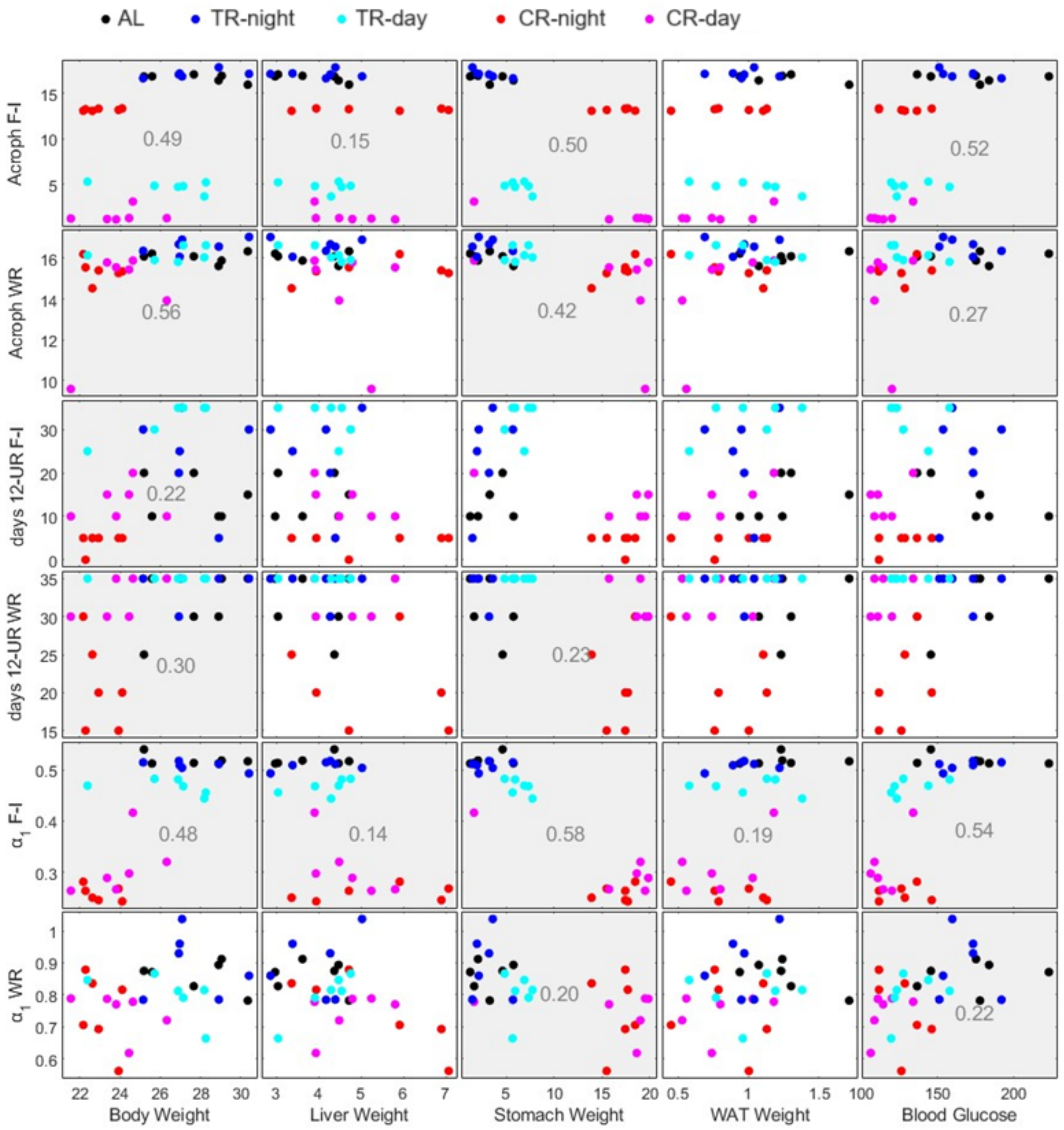

Supplementary Figure 21. Correlation coefficient estimates between behavioral dynamic variables and physiological variables in mice under different feeding paradigms. Each circle represents an individual, with the color denoting the treatment as indicated in the legend key. Light grey backgrounds indicate statistically significant correlations ($P<0.05$) with grey numbers indicating the value of the coefficient of determination. Food-intake (F-I) and Wheel running (WR) acrophase associated with the circadian rhythm are the same as data shown in Supplementary Figure 10. The number of days in which 12h URs were observed in each behavior is the same as shown in Figure 2q and L, as with α-values in Figure 3c and d.

Supplementary Table 1. Autovectors of the Principal Component Analysis of behavioral dynamics associated with Figure 4a.

Variable	e1	e2
24h-rhythm acrophase Food-intake	0.05	0.78
24h-rhythm acrophase Wheel running	0.50	0.51
Days with 12h rhythms Food-intake	0.65	-0.16
Days with 12h rhythms Wheel running	0.57	-0.33

Supplementary Table 2. Correlations between behavioral dynamics variables and the first two principal components (PC1 and PC2) of the PCA associated with Figure 4a.

Variable	PC1	PC2
24h-rhythm acrophase Food-intake	0.07	0.91
24h-rhythm acrophase Wheel running	0.67	0.59
Days with 12h rhythms Food-intake	0.87	-0.19
Days with 12h rhythms Wheel running	0.77	-0.39

Supplementary Table 3. Autovectors of the Principal Component Analysis of behavioral dynamics variables associated with Figure 4b.

Variable	e1	e2
24h-rhythm acrophase Food-intake	0.25	0.71
24h-rhythm acrophase Wheel running	0.39	0.21
α1 Food-intake	0.55	0.03
α1 Wheel running	0.39	0.27
Days with 12h rhythms Food-intake	0.43	-0.39
Days with 12h rhythms Wheel running	0.39	-0.49

Supplementary Table 4. Correlations between behavioral dynamics and physiological variables and the first two principal components (PC1 and PC2) of the PCA associated with Figure 4B.

Variable	PC1	PC2
24h-rhythm acrophase Food-intake	0.41	0.86
24h-rhythm acrophase Wheel running	0.66	0.25
α1 Food-intake	0.92	0.03
α1 Wheel running	0.66	0.27
Days with 12h rhythms Food-intake	0.73	-0.47
Days with 12h rhythms Wheel running	0.65	-0.59

Supplementary Table 5. Autovectors of the Principal Component Analysis of behavioral dynamics and physiological variables associated with Figure 4C.

Variable	e1	e2
24h-rhythm acrophase Food-intake	0.24	0.64
24h-rhythm acrophase Wheel running	0.30	0.10
Body weight	0.38	-0.06
Liver weight	-0.31	0.20
Blood glucose	0.34	0.36
α1 Food-intake	0.46	-0.01
α1 Wheel running	0.31	0.16
Days with 12h rhythms Food-intake	0.31	-0.42
Days with 12h rhythms Wheel running	0.31	-0.45

Supplementary Table 6. Correlations between behavioral dynamics and physiological variables and the first two principal components (PC1 and PC2) of the PCA associated with Figure 4c.

Variable	PC1	PC2
24h-rhythm acrophase Food-intake	0.50	0.82
24h-rhythm acrophase Wheel running	0.62	0.13
Body weight	0.79	-0.08
Liver weight	-0.63	0.25
Blood glucose	0.71	0.46
α1 Food-intake	0.94	-0.02
α1 Wheel running	0.63	0.20
Days with 12h rhythms Food-intake	0.63	-0.54
Days with 12h rhythms Wheel running	0.64	-0.58

Supplementary Table 7. Autovectors of the Principal Component Analysis of physiological variables associated with Figure 4d.

Variable	e1	e2
Body weight	0.50	-0.03
Liver weight	0.36	0.75
Blood glucose	-0.43	0.16
Stomach weight	0.55	0.04
WAT weight	-0.37	0.64

Supplementary Table 8. Correlations between physiological variables and the first two principal components (PC1 and PC2) of the PCA associated with Figure 4d.

Variable	PC1	PC2
Body weight	-0.84	-0.03
Liver weight	0.60	0.73
Blood glucose	-0.72	0.15
Stomach weight	0.93	0.04
WAT weight	-0.63	0.62

Supplementary References

1. Flesia AG, Nieto PS, Aon MA, Kembro JM. Computational Approaches and Tools as Applied to the Study of Rhythms and Chaos in Biology. Methods Mol Biol. 2022;2399:277-341. doi: 10.1007/978-1-0716-1831-8_13. PMID: 35604562

2. Kembro JM, Flesia AG, Nieto PS, Caliva JM, Lloyd D, Cortassa S, Aon MA. A dynamically coherent pattern of rhythms that matches between distant species across the evolutionary scale. Sci Rep. 2023 Apr 1;13(1):5326. doi: 10.1038/s41598-023-32286-0. PMID: 37005423; PMCID: PMC10067965.

3. Acosta-Rodríguez VA, de Groot MHM, Rijo-Ferreira F, Green CB, Takahashi JS. Mice under Caloric Restriction Self-Impose a Temporal Restriction of food-intake as Revealed by an Automated Feeder System. Cell Metab. 2017 Jul 5;26(1):267-277.e2. doi: 10.1016/j.cmet.2017.06.007. PMID: 28683292; PMCID: PMC5576447.
4. Acosta-Rodríguez VA, Rijo-Ferreira F, Green CB, Takahashi JS. Importance of circadian timing for aging and longevity. Nat Commun. 2021 May 17;12(1):2862. doi: 10.1038/s41467-021-22922-6. PMID: 34001884; PMCID: PMC8129076.

5. Holmes, M. M. & Mistlberger, R. E. Food anticipatory activity and photic entrainment in food-restricted BALB/c mice. Physiol. Behav. 68, 655–666 (2000)

6. Castillo, M. R. et al. Entrainment of the master circadian clock by scheduled feeding. Am. J. Physiol. Regul. Integr. Comp. Physiol. 287, R551–R555 (2004).

7. Damiola F, Le Minh N, Preitner N, Kornmann B, Fleury-Olela F, Schibler U. Restricted feeding uncouples circadian oscillators in peripheral tissues from the central pacemaker in the suprachiasmatic nucleus. Genes Dev. 2000 Dec 1;14(23):2950-61. doi: 10.1101/gad.183500. PMID: 11114885; PMCID: PMC317100.

8. Stokkan KA, Yamazaki S, Tei H, Sakaki Y, Menaker M. Entrainment of the circadian

clock in the liver by feeding. Science. 2001 Jan 19;291(5503):490-3. doi: 10.1126/science.291.5503.490. PMID: 11161204.

9. Challet, E. The circadian regulation of food-intake. *Nat Rev Endocrinol* **15**, 393–405 (2019).

10. Mistlberger RE. Neurobiology of food anticipatory circadian rhythms. Physiol Behav. 2011 Sep 26;104(4):535-45. doi: 10.1016/j.physbeh.2011.04.015. Epub 2011 Apr 20. PMID: 21527266.

11. Challet E, Solberg LC, Turek FW. Entrainment in calorie-restricted mice: conflicting zeitgebers and free-running conditions. Am J Physiol. 1998 Jun;274(6):R1751-61. doi: 10.1152/ajpregu.1998.274.6.R1751. PMID: 9841486.

12. Cambras T, Vilaplana J, Díez-Noguera A. Effects of long-term restricted feeding on motor activity rhythm in the rat. Am J Physiol. 1993 Aug;265(2 Pt 2):R467-73. doi: 10.1152/ajpregu.1993.265.2.R467. PMID: 8368403.

13. Andrade JP, Pereira PA, Silva SM, Sá SI, Lukoyanov NV. Timed hypocaloric food restriction alters the synthesis and expression of vasopressin and vasoactive intestinal peptide in the suprachiasmatic nucleus. Brain Res. 2004 Oct 1;1022(1-2):226-33. doi: 10.1016/j.brainres.2004.07.013. PMID: 15353233.

14. Mendoza J, Angeles-Castellanos M, Escobar C. A daily palatable meal without food deprivation entrains the suprachiasmatic nucleus of rats. Eur J Neurosci. 2005 Dec;22(11):2855-62. doi: 10.1111/j.1460-9568.2005.04461.x. PMID: 16324120.

15. Mendoza J, Pévet P, Challet E. Circadian and photic regulation of clock and clock-controlled proteins in the suprachiasmatic nuclei of calorie-restricted mice. Eur J Neurosci. 2007 Jun;25(12):3691-701. doi: 10.1111/j.1460-9568.2007.05626.x. PMID: 17610588.

16. Resuehr D, Olcese J. Caloric restriction and melatonin substitution: effects on murine

circadian parameters. Brain Res. 2005 Jun 28;1048(1-2):146-52. doi: 10.1016/j.brainres.2005.04.063. PMID: 15913571.

17. Challet E. Interactions between light, mealtime and calorie restriction to control daily timing in mammals. J Comp Physiol B. 2010 Jun;180(5):631-44. doi: 10.1007/s00360-010-0451-4. Epub 2010 Feb 20. PMID: 20174808.

18. Peng CK, Havlin S, Stanley HE, Goldberger AL. Quantification of scaling exponents and crossover phenomena in nonstationary heartbeat time series. Chaos. 1995;5(1):82-7. doi: 10.1063/1.166141. PMID: 11538314.

19. Goldberger AL, Amaral LA, Hausdorff JM, Ivanov PCh, Peng CK, Stanley HE. Fractal dynamics in physiology: alterations with disease and aging. Proc Natl Acad Sci U S A. 2002 Feb 19;99 Suppl 1(Suppl 1):2466-72. doi: 10.1073/pnas.012579499. PMID: 11875196; PMCID: PMC128562.

20. Peng CK, Buldyrev SV, Havlin S, Simons M, Stanley HE, Goldberger AL. Mosaic organization of DNA nucleotides. Phys Rev E 1994 Feb;49(2):1685-9. doi: 10.1103/physreve.49.1685. PMID: 9961383.

21. Maraun, D, Rust, HW, Timmer, J. Tempting long-memory – on the interpretation of DFA results. Nonlinear Proc Geoph. 2004 11:495-503. doi: 10.5194/npg-11-495-2004.

22. Hu K, Ivanov PC, Chen Z, Carpena P, Stanley HE. Effect of trends on detrended fluctuation analysis. Phys Rev E Stat Nonlin Soft Matter Phys. 2001 Jul;64(1 Pt 1):011114. doi: 10.1103/PhysRevE.64.011114. Epub 2001 Jun 26. PMID: 11461232.

23. Kembro, JM, Flesia, AG, Gleiser, RM, Perillo, MA, Marin, RH. Assessment of long-range correlation in animal behavior time series: The temporal pattern of locomotor activity of Japanese quail (*Coturnix coturnix*) and mosquito larva (*Culex quinquefasciatus*). Phys A 2013:392:6400-6413. doi: https://doi.org/10.1016/j.physa.2013.08.017.

24. Macintosh AJ, Pelletier L, Chiaradia A, Kato A, Ropert-Coudert Y. Temporal fractals in seabird foraging behaviour: diving through the scales of time. Sci Rep. 2013;3:1884. doi: 10.1038/srep01884. PMID: 23703258; PMCID: PMC3662970.
