# Supplementary material for: Dietary restriction modulates ultradian rhythms and autocorrelation properties in mice behavior
Source: Commun Biol. 2024 Mar 9;7:303. doi: 10.1038/s42003-024-05991-3 (PMC10925031; doi:10.1038/s42003-024-05991-3)
Supplement: Supplementary file 3 — Description of Additional Supplementary Files [file 42003_2024_5991_MOESM3_ESM.pdf]

Description of Additional Supplementary Files

File name: Supplementary Data

Description: The source data behind the Figures 1-3 in the paper.
